# Autistic adults benefit from and enjoy learning via social interaction as much as neurotypical adults do

**DOI:** 10.1186/s13229-023-00561-6

**Published:** 2023-09-06

**Authors:** S. De Felice, A. Hatilova, F. Trojan, I. Tsui, Antonia F. de C. Hamilton

**Affiliations:** Institute of Cognitive Neuroscience, University College London, Alexandra House, 17-19 Queen Square, London, WC1N 3AZ UK

**Keywords:** Social learning, Social cognition, Social interaction, Autism, Online education

## Abstract

**Background:**

Autistic people show poor processing of social signals (i.e. *about* the social world). But how do they learn via social interaction?

**Methods:**

68 neurotypical adults and 60 autistic adults learned about obscure items (e.g. exotic animals) over Zoom (i) in a live video-call with the teacher, (ii) from a recorded learner-teacher interaction video and (iii) from a recorded teacher-alone video. Data were analysed via analysis of variance and multi-level regression models.

**Results:**

Live teaching provided the most optimal learning condition, with no difference between groups. Enjoyment was the strongest predictor of learning: both groups enjoyed the live interaction significantly more than other condition and reported similar anxiety levels across conditions.

**Limitations:**

Some of the autistic participants were self-diagnosed—however, further analysis where these participants were excluded showed the same results. Recruiting participants over online platforms may have introduced bias in our sample. Future work should investigate learning in social contexts via diverse sources (e.g. schools).

**Conclusions:**

These findings advocate for a distinction between learning *about* the social versus learning via the social: cognitive models of autism should be revisited to consider social interaction not just as a puzzle to decode but rather a medium through which people, including neuro-diverse groups, learn about the world around them.

*Trial registration* Part of this work has been pre-registered before data collection https://doi.org/10.17605/OSF.IO/5PGA3

**Supplementary Information:**

The online version contains supplementary material available at 10.1186/s13229-023-00561-6.

## Introduction

We live in a world that is more digital by the day. With the rise in online learning, which has seen an impetus since the Covid-19 pandemic, it is important to understand what social contexts best support learning across a wide range of populations. We have recently shown that social interaction boosts learning in neurotypical (NT) adults [17]. Here, we ask whether the same live-learning advantage would be replicated in a sample of autistic adults (for autism language use throughout the paper we refer to the work from [36] and follow on reflections and suggestions from [45].

Autism is a neurodevelopmental condition manifesting in infancy or early childhood [1]. While the specific autistic experiences and characteristics can vary significantly across individuals [30]—leading to the condition being identified as a ‘spectrum’ (e.g. [8], difficulties in communication and social interaction are central to the diagnosis of autism [1]. These include implicit imitation [6, 21], joint attention [46, 52], social perception [57], pragmatic language use [62] and affect sharing [30]. All these differences in aspects of social communication and social interaction hint at the possibility that autistic people might find it hard to learn new information in a social context.

A number of studies show that autistic children show less spontaneous imitation behaviour [21, 27, 63, 42], which suggests that they may find it hard to learn via imitation. Differences in social attention in autism might also lead to differences in social learning [40, 48, 49, 51, 53, 56]. A large meta-analysis of 122 studies confirmed a reliable pattern of gaze differences in autistic individuals that persisted across ages, specifically in selecting socially relevant versus irrelevant information for attention, especially during perception of social interactions [25]. However, recent work on children suggests that these conclusions may be restricted to lab-based artificial experiments, and may not extend to naturalistic interactions [66]. Autistic people may also have difficulty in learning to use some types of social information, resulting in poor labelling of facial emotions [58]. However, other types of social information are learnt normally, including social stereotypes [33]. Thus, it is not yet clear whether and how difficulties in some aspects of social cognition in autism impact their ability to learn in the context of social interaction.

The motivation to engage socially is a second factor which might impact on learning in autistic people. Clinical and experimental observations suggest that autistic people show disrupted processing of social rewards [13, 64] and less flexible behaviour [54]. The social motivation hypothesis argues that autistic people engage less in social contexts as they do not find these rewarding [9, 10, 14, 20, 47]. However, others have argued against the idea that autism presents social reward difficulty (see [34, 44, 64]), so the topic of social motivation in autism remains controversial [7]. In relation to social learning, reward and enjoyment are a key factor that drive many people to engage in a variety of types of learning, but it is not clear if these are differentially affected in autism. By measuring enjoyment of learning during a social learning task, we hope to gain a better understanding of whether motivation to learn socially might differ in autism.

Taken together, previous studies imply that autistic people may fail to show an interactive-learning advantage. This could be either due to general difficulties in social cognition or to a reduction in social motivation or to differences in social attention. However, previous studies of learning in autism have mostly looked at *implicit* learning of *social* material in relatively constrained experiments (e.g. facial-emotion labelling, [58], language, [24, 55]). We are not aware of studies which look at *explicit* learning of *non-social material* where social interaction provides a medium via which the material can be learnt. Thus, what remains unclear is whether the acquisition of *non-social* knowledge benefits from *social interaction* equally in autistic people as in NT during naturalistic interactions. To the best of our knowledge, this question still needs to be explored empirically.

In the present study, we compare learning performance during three different social learning conditions (one live and two recorded), where learning content is always delivered online by a (human) teacher (similar to [17]). In all conditions, participants are explicitly instructed to learn facts about unfamiliar items (e.g. exotic animals). In the *live* condition, the participant joins a live video-call where they can interact with the teacher. In the *recorded-observant* condition, the participant learns the material from a pre-recorded video of a previous session (observing a previously recorded learner-teacher interaction). In the *recorded-alone* condition, the participant learns the material from a pre-recorded video of the teacher alone. In order to relate learning to motivational factors, we include measures of enjoyment, and we also include measures of autistic traits and verbal abilities.

We expect to replicate results from our previous study [17], showing a live-learning advantage in NT adults. Regarding autistic people, according to the literature showing social cognition and social motivation differences, one could speculate that no advantage in the live condition would be observed in this group. However, this speculation would be based on studies which mainly looked at implicit learning of social content, in contrast to declarative interactive learning of non-social knowledge. We, therefore, do not have strong predictions about performance in the autistic group. We first present results from a pilot study and then show findings from a larger pre-registered replication.

## Experiment 1

### Methods

#### Design

**Fig. 1:**
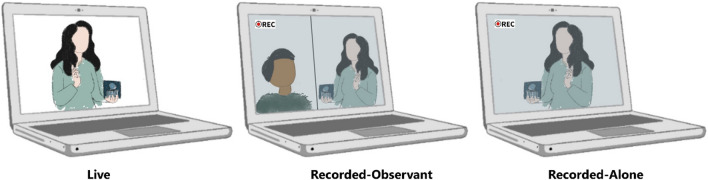
Schematics of the three experimental conditions

This study aims to investigate whether (i) participating in a live learning session improves learning online compared to recorded videos of either a previous interaction or of a teacher alone and (ii) whether these conditions impact learning differently in autistic adults compared to neurotypicals. To answer these questions, this study adopted a 2 (group) × 3 (learning condition) × 2 (time) repeated-measures design, with between- and within-subjects factors. The between-subjects factor is group (autistic vs neurotypical (NT)), the within-subjects factors are (i) learning condition (live vs recorded of another social learning episode vs recorded of the teacher alone), and (ii) time of recall (immediate vs delay quiz). Specifically, facts about 15 items were presented with two minutes per item. Five items were assigned to each condition: (1) live condition: the participant participated in a live video-call when they learned in interaction with the teacher; (2) recorded-observant condition: the participant was shown a pre-recorded video of the teacher presenting the learning material to a student (confederate); (3) recorded alone condition: the participant was shown a pre-recorded video of the teacher alone presenting the learning material (Fig. 1). The learning score (outcome measure) for each participant was obtained from a multiple-choice quiz (see Materials). Items assigned to each condition and trial order within each condition remained fixed for the whole duration of this experiment. The order of conditions was randomised across participants.

#### Materials

A selection of 15 items was selected from De Felice et al. [17], three from the exotic food category (*Rambutan, Kiwano, Cherimoya*), four from the antique category (*Strigil, Porte-joupe, Scotch Hands, Chatelaine*), four from the animal category (*Tarsier, Axolotl, Glaucus, Anhinga*) and four from the musical instrument category (*Kalimba, Caxixi, Agogo, Hulusi*). Each condition presented a mixture of objects from these categories, which remained fixed for all participants for this experiment (but was counterbalanced in experiment 2, see Sect. 3), as follows: (1) live condition [*Tarsier, Kalimba, Strigil, Axolotl, Rambutan*]; (2) recorded-observant condition [*Porte-joupe, Kiwano, Caxixi, Scotch Hands, Glaucus*]; (3) recorded-alone condition [*Agogo, Cherimoya, Anhinga, Chatelaine, Hulusi*]. Learning was tested via the same multiple-choice quiz used by De Felice et al. [17]. Full details of item information and multiple-choice quiz are reported in Additional file 1: Appendix Table 1.

Learning sessions are represented as appearing to participants. From left to right: In the live condition, participants learn about five items as they interact with the teacher in a real-time video-call; Recorded-observant condition: participants learn about five items from a pre-recorded sessions with a confederate acting as a previous participant; Recorded-Alone condition: participants learn about five items from a pre-recorded session of the teacher alone. In each condition, participants learn about five different items. Items were assigned to each condition randomly and remained fixed within each experiment, and counterbalanced between experiments.

#### Procedure

*Participant recruitment* This study was approved by the UCL ethics committee. Participants were recruited via the online platform Prolific (www.prolific.co). The platform retains demographic details as well as information on any disabilities/diagnoses of users, as reported by the users at the time of account registration. Such anonymous information can be used to create adverts targeting a specific pool. Two separate adverts were published: one targeting neurotypical participants and one targeting autistic participants. As a further check, users who responded to our adverts were asked to confirm their diagnosis via a questionnaire on Gorilla Experiment Builder (www.gorilla.sc). To ensure that the experimenter was blind to the participant’s diagnosis, recruitment was done by a researcher who was not involved in data collection.

To be eligible, all participants had to (i) be fluent in English (speaking English regularly for > 5 years); (ii) be aged 18–65; (iii) give consent to having their camera and microphone on; and (iv) give consent to being recorded for the whole duration of the experiment. Participants were paid at the hourly rate of £7.50 for a total of £15 over two hours. An additional £3 was offered for those who completed a 10 min quiz a week later.

Participants who responded to our advert were asked to complete four main tasks: (1) background battery (independently online, on Gorilla Experiment Builder), (2) learning session (over a video-call), (3) online learning multiple-choice quiz immediately after the learning session (independently online on Gorilla Experiment Builder), and (4) repeat the quiz a week later.

*Background battery* Users who responded to the Prolific adverts were redirected to Gorilla Experiment Builder (www.gorilla.sc), where they received instructions on the study and gave consent for participation. They then completed the Background Battery tasks. This comprises of (i) Spot-the-word test, a measure of verbal fluency [3], (ii) matrix reasoning item bank (MaRs-IB), a measure of non-verbal reasoning [11] and (iii) Animated Triangle test, a measure of mentalising [2, 61].

Upon completion of the Background battery task, an independent researcher sent the participant ID to the experimenter (teacher), who arranged a video-call with the participant (via Prolific chat) while remaining blind to their diagnosis.

*Video-call* The experimenter greeted the participant and checked that audio and video worked adequately. The participant was asked to open the zoom window in full-screen mode and chose the gallery view (i.e. everyone in the call is shown equal size, this ensured that view during the live session was comparable to the view during pre-recorded video watching). Participants were told that the aim of the study was to investigate how people learned online and whether this differed in autistic people. They were asked not to disclose personal information to the teacher, who was blind to their diagnosis. The experimenter then explained that the participant would learn some facts about exotic food, animals, antiques and rare musical instruments over three formats: in live interaction with the teacher (live condition) and through watching pre-recorded videos showing either the teacher with a previous participant (recorded-observant condition) or the teacher alone (recorded-alone condition). They were instructed to memorise as much information as possible, as at the end of the video-call, they would complete a multiple-choice quiz to test their learning. During the live condition, participants were told they were free to ask questions and interact with the teacher. Before starting the learning sessions, the participant’s pre-knowledge was tested. If any item was known, this was excluded from the analysis (but not from the learning session). Learning sessions started with either the live, recorded-alone or recorded-observant condition in a counterbalanced and semi-randomised order. The call lasted approximately 40 min (i.e., 10 min per condition, with 2 min per item and five items in each condition, plus 10 min for instructions).

*Learning quiz* Immediately after the learning session, participants were redirected to Prolific, where their IDs were included in a ‘white list’ so that a new advert was visible to them only. By replying to that advert, participants were redirected to Gorilla Experiment Builder (www.gorilla.sc), where they reported on the quality of the video call (audio and video) before completing the learning quiz. After the learning quiz, they also completed an ‘enjoyment questionnaire’ and the inclusion-of-other-in-the-self questionnaire (Aron et al., 1992). This part lasted approximately 20 min and was completed by the participant independently (note that the ‘immediateness’ of the quiz was ensured by the experimenter, who terminated the video-call only a few moments after the participant initiated the quiz part on Gorilla Experimenter Builder).

Exactly one week after the learning sessions, participants were invited through Prolific to the final stage of the experiment and directed to Gorilla Experimenter Builder to complete the same learning quiz. Additionally, participants filled in a history questionnaire to check for potential revision of any of the items (e.g. search on Google). This part lasted approximately 10 min.

### Results

#### Sample

53 participants took part in the study (Table 1). Participants were excluded when reporting 3 or less on a 1 (poor) to 5 (excellent) video-call quality scale (N = 3), and being visibly distracted during the video-call (N = 1). Of the remaining 49 participants, 46 (N_autistic_ = 20, male = 11, female = 4, non-binary = 4; N_NT_ = 26, male = 11, female = 15) completed the whole experiment, including the one-week delay quiz (see 2.1 Design and 2.3 Procedure). We lost demographic data from one participant (autistic group) due to a technical fault.

**Table 1:** Demographics for the dataset from Experiment 1, 2 and combined

Experiment 1 (N = 46)	Neurotypical (NT) group N = 26		Autistic group N = 20		NT versus Autistic group	
	Mean	SD	Mean	SD	t-test	*p*-value
Age	29.85	9.90	27.79	9.22	0.71	*0.48*
Verbal fluency (Spot the word)^1^	44.73	6.23	47.63	6.71	− 1.49	*0.14*
Non-verbal reasoning (Matrix reasoning item bank, MaRs-IB)^2^	61.64	16.98	64.48	18.57	− 0.52	*0.60*
Mentalising (Animated Triangle)^3^	9.62	1.87	8.74	2.35	0.88	*0.18*
Autistic Quotient (AQ)^4^	19.19	7.29	33.37	6.73	− 6.65	< *0.0001*

Experiment 2 (N = 82)	Neurotypical group (NT) N = 42		Autistic group N = 40		NT versus Autistic group	
	Mean	SD	Mean	SD	t-test	*p*-value
Age	27.50	4.91	27.63	5.13	0.13	*0.89*
Verbal fluency (Spot the word)^1^	44.54	8.46	45.73	9.83	− 0.59	*0.56*
Non-verbal reasoning (Matrix reasoning item bank, MaRs-IB)^2^	61.37	17.26	63.08	18.68	− 0.43	*0.67*
Mentalising (Animated Triangle)^3^	8.93	2.22	9.37	1.85	− 0.44	*0.33*
Autistic Quotient (AQ)^4^	19.98	6.74	28.39	8.60	− 4.93	< *0.0001*

Combined (N = 128)	Neurotypical (NT) group N = 68		Autistic group N = 60		NT versus Autistic group	
	Mean	SD	Mean	SD	t-test	*p*-value
Age	28.49	7.27	27.58	6.62	0.73	*0.46*
Verbal fluency (Spot the word)^1^	44.61	7.63	46.33	8.95	− 1.17	*0.24*
Non-verbal reasoning (Matrix reasoning item bank, MaRs-IB)^2^	61.47	17.02	63.52	18.50	− 0.65	*0.51*
Mentalising (Animated Triangle)^3^	9.19	2.10	9.17	2.03	0.08	*0.94*
Autistic Quotient (AQ)^4^	19.67	6.91	29.97	8.33	− 7.60	< *.0001*

Sample size (N), age, AQ score and performance on background battery tests for neurotypical (NT) and Autistic groups. Note that the higher the AQ score, the greater the autistic traits. 1. Baddeley et al. [3] 2. Chierchia et al. [11] 3. Livingston et al. [40] 4. Baron-Cohen et al. [4]

Italic values indicate for p-values

Autistic participants either received a diagnosis by a clinician (N = 18) or were self-diagnosed (N = 2). Autistic and NT groups did not differ on age (mean_autistic_ (sd) = 27.79 (9.22), mean_NT_ (sd) = 29.85 (9.90), t_(43)_ = 0.71, *p* = 0.48), verbal fluency (Spot the word test, mean_autistic_ (sd) = 47.63 (6.71), mean_NT_ (sd) = 44.73 (6.23), t_(43)_ = − 1.48, *p* = 0.15) non-verbal reasoning (MaRs-IB, mean_autistic_ (sd) = 64.48 (18.57), mean_NT_ (sd) = 61.64 (16.98), t_(43)_ = − 0.52, *p* = 0.60) and mentalising test (Animated Triangle, mean_autistic_ (sd) = 9 (2.54), mean_NT_ (sd) = 9.33 (1.92), t_(43)_ = 0.38, *p* = 0.71). The autistic group scored significantly higher on AQ than the NT group (mean_autistic_ (sd) = 33.37 (6.73), mean_NT_ (sd) = 19.19 (7.29); t_(43)_ = − 6.73, *p* < 0.0001).

#### Data pre-processing

Single trials were excluded when: (i) participants reported that they knew the item; (ii) internet connection dropped during the single trial but was good for the rest of the experiment; (iii) the experimenter reported incorrect information about the item; (iv) participants reported revising information about a given item before the delay quiz (excluded from delay performance only). Performance was calculated for each learning condition separately, as an average over the included trials (score = points collected on all trials / total points available on all included trials).

#### Analysis of variance

**Fig. 2:**
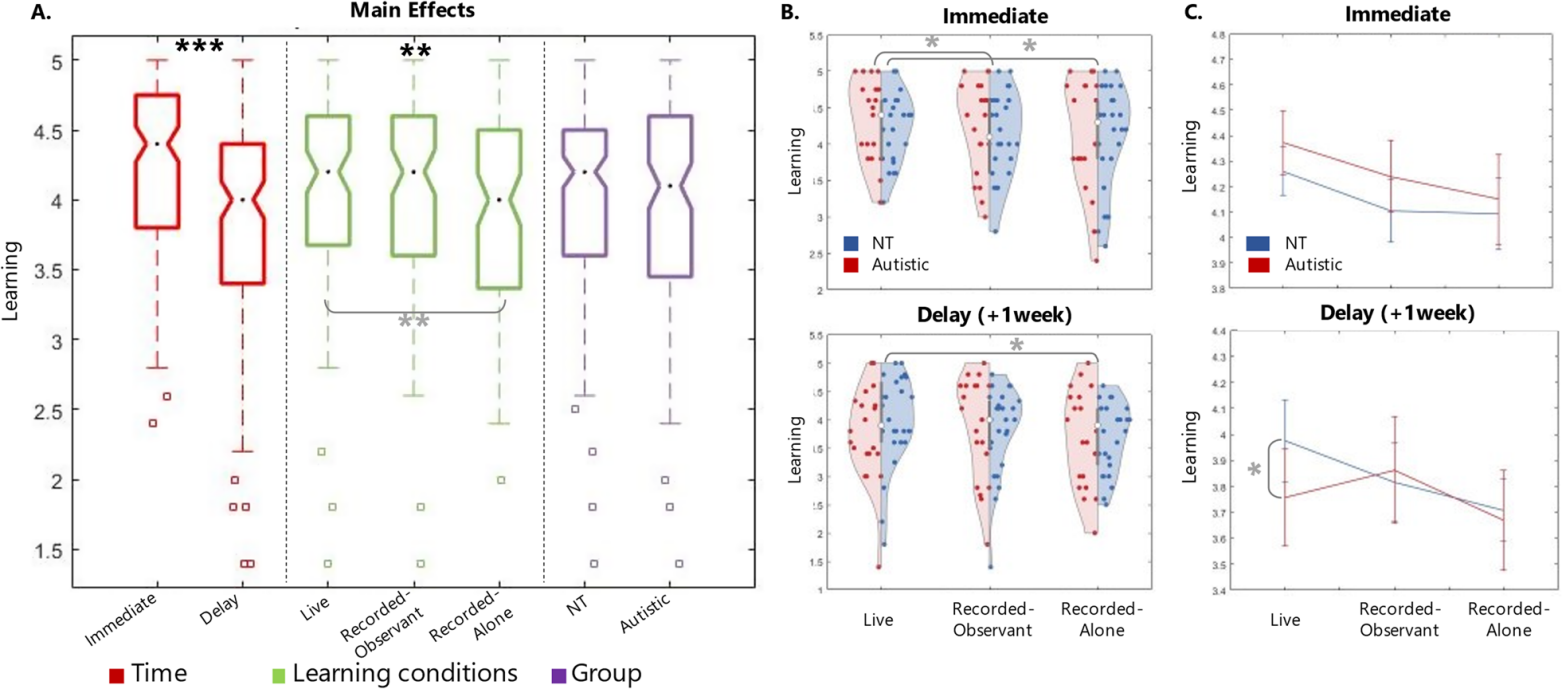
Results for Experiment 1. Results from the dataset of Experiment 1. *p < 0.05; **p < 0.001;***p < 0.0001. **A** Boxplots of the three main factors of interest: Time, Learning condition and Group. **B** Violin plots of learning performance immediately after the learning session (top) and a week later (bottom) for the three learning conditions. Violins are split in half, showing the distribution of NT (blue) and Autistic (red) samples separately. **C** Line plot for learning performance immediately after the learning session (top) and after one week (bottom), plotted separately for NT (blue) and Autistic group (red). Error bars represent the standard error of the mean

An analysis of variance (ANOVA) was run to test the difference in learning performance between 2 (groups: Autistic and NT) × 3 (learning conditions: live, recorded-alone and recorded-observant) × 2 (time of learning quiz: immediate and delay). Means and SD for all conditions are reported in Table 2. Results for main and interaction effects are reported in Table 3 and Fig. 2.

**Table 2:** Sample size (N), Means and SDs for all conditions for Experiment 1, 2 and combined

	N	Mean	SD
*Experiment 1—Recall scores*			
**Neurotypical (NT) group**			
Immediate: Live	26	4.26	0.10
Immediate: Recorded-observant	26	4.10	0.12
Immediate: Recorded-alone	26	4.09	0.15
+ 1 week: Live	26	3.98	0.16
+ 1 week: Recorded-observant	26	3.82	0.16
+ 1 week: Recorded-alone	26	3.71	0.14
**Autistic group**			
Immediate: Live	20	4.37	0.12
Immediate: Recorded-observant	20	4.24	0.14
Immediate: Recorded-alone	20	4.15	0.17
+ 1 week: Live	20	3.75	0.18
+ 1 week: Recorded-observant	20	3.86	0.19
+ 1 week: Recorded-alone	20	3.67	0.16
*Experiment 2—Recall scores*			
**Neurotypical (NT) group**			
Immediate: Live	41	4.33	0.08
Immediate: Recorded-observant	41	3.93	0.10
Immediate: Recorded-alone	41	4.15	0.10
+ 1 week: Live	41	3.84	0.12
+ 1 week: Recorded-observant	41	3.58	0.13
+ 1 week: Recorded-alone	41	3.75	0.14
**Autistic group**			
Immediate: Live	41	4.46	0.08
Immediate: Recorded-observant	41	4.13	0.10
Immediate: Recorded-alone	41	4.33	0.10
+ 1 week: Live	41	4.01	0.13
+ 1 week: Recorded-observant	41	3.81	0.13
+ 1 week: Recorded-alone	41	4	0.14
*Combined—Recall scores*			
**Neurotypical (NT)** **group**			
Immediate: Live	67	4.30	0.06
Immediate: Recorded-observant	67	4	0.08
Immediate: Recorded-alone	67	4.13	0.08
+ 1 week: Live	67	3.89	0.10
+ 1 week: Recorded-observant	67	3.67	0.10
+ 1 week: Recorded-alone	67	3.75	0.10
**Autistic** **group**			
Immediate: Live	61	4.43	0.07
Immediate: Recorded-observant	61	4.16	0.08
Immediate: Recorded-alone	61	4.27	0.09
+ 1 week: Live	61	3.93	0.10
+ 1 week: Recorded-observant	61	3.83	0.11
+ 1 week: Recorded-alone	61	3.89	0.11

Mean number of items recalled at test (max of 5) for each condition for neurotypical (NT) and Autistic group for each Experiment and for combined datasets

**Table 3:** Results for analysis of variance for Experiment 1, 2 and combined datasets

Comparisons	F or T value	df	*p*	Partial Eta Squared	95% CI	
					Lower bound	Upper bound
Experiment 1 (N = 46)	F					
*Main effects*						
Group	0.007	1	*0.93*	0.00	− 0.39	0.36
Learning condition	4.06	2	***0.016***	0.09	–	–
Time	56.16	1	** < *0.0001***	0.56	0.30	0.51
*Interaction effects*	F					
Group*Time	2.56	1	*0.12*	0.06	–	–
Group*Learning condition	0.62	2	*0.54*	0.01	–	–
Learning condition*Time	0.92	2	*0.40*	0.02	–	–
Learning condition*Time*Group	1.08	2	*.34*	0.02	–	–

	t	df	*p*	Partial Eta Squared	CI Lower bound	CI Upper bound
*Simple effects*						
Live versus Recorded-observant	0.08	45	*0.14*	–	− 0.03	0.2
Live versus Recorded-alone	0.19	45	***0.008***	–	0.05	0.32
Recorded-observant versus Recorded-alone	0.10	45	*0.16*	–	− 0.04	0.24
Live_imm_ versus Recorded-observant_imm_	2.51	45	***0.016***	–	0.03	0.26
Live_imm_ versus Recorded-alone_imm_	2.41	45	***0.02***	–	0.03	0.35
Recorded-observant_imm_ versus Recorded-alone_imm_	0.62	45	*0.53*	–	− 0.10	0.19
Live_del_ versus Recorded-observant_del_	0.54	45	*0.59*	–	− 0.11	0.20
Live_del_ versus Recorded-alone_del_	2.13	45	***0.04***	–	0.01	0.37
Recorded-observant_del_ versus Recorded-alone_del_	1.56	45	*0.12*	–	− 0.04	0.33

Experiment 2 (N = 82)	F	df	*p*	Partial Eta Squared	CI Lower bound	CI Upper bound
*Main effects*						
Group	2.24	1	*0.14*	0.03	− 0.45	0.06
Learning condition	13.54	2	** < *0.0001***	0.14	–	–
Time	38.56	1	** < *0.0001***	0.33	0.26	0.51
*Interaction effects*	F					
Group*Time	0.16	1	*0.69*	0.002	–	–
Group*Learning condition	0.18	2	*0.83*	0.002	–	–
Learning condition*Time	2.12	2	*0.12*	0.03	–	–
Learning condition*Time*Group	0.03	2	*0.97*	0.00	–	–

	t	df	*p*	Partial Eta Squared	CI Lower bound	CI Upper bound
*Simple effects*						
Live versus Recorded-observant	0.30	1	** < *0.0001***	–	0.18	0.41
Live versus Recorded-alone	0.10	1	*0.09*	–	− 0.02	0.22
Recorded-observant versus Recorded-alone	− 0.19	1	***0.001***	–	− 0.31	− 0.08
Live_imm_ versus Recorded-observant_imm_	5.86	45	** > *0.0001***	–	0.24	0.49
Live_imm_ versus Recorded-alone_imm_	2.32	45	***0.02***	–	0.02	0.28
Recorded-observant_imm_ versus Recorded-alone_imm_	− 3.21	45	***0.002***	–	− 0.34	− 0.08
Live_del_ versus Recorded-observant_del_	3.32	45	***0.001***	–	0.09	0.36
Live_del_ versus Recorded-alone_del_	0.74	45	*0.46*	–	− 0.08	0.18
Recorded-observant_del_ versus Recorded-alone_del_	− 2.49	45	***0.01***	–	− 0.32	− 0.04

Combined (N = 128)	F	df	*p*	Partial Eta Squared	CI Lower bound	CI Upper bound
*Main effects*						
Group	1.54	1	*0.22*	0.01	− 0.34	0.08
Learning condition	12.63	2	** < *0.0001***	0.09	–	–
Time	77.32	1	** < *0.0001***	0.38	0.30	0.49
*Interaction effects*	**F**					
Group*Time	0.11	1	*0.75*	0.001	–	–
Group*Learning condition	0.45	2	*0.64*	0.004	–	–
Learning condition*Time	2.61	2	*0.07*	0.02	–	–
Learning condition*Time*Group	0.5	2	*0.6*	0.004	–	–

	t	df	*p*	Partial Eta Squared	CI Lower bound	CI Upper bound
*Simple effects*						
Live versus Recorded-observant	0.22	1	** < *0.0001***	–	0.14	0.31
Live versus Recorded-alone	0.13	1	***0.004***	–	0.04	0.22
Recorded-observant versus Recorded-alone	− 09	1	***0.05***	–	0.18	0.002
Live_imm_ versus Recorded-observant_imm_	6.24	127	** < *0.0001***	–	0.19	0.37
Live_imm_ versus Recorded-alone_imm_	3.28	127	***0.001***	–	0.07	0.27
Recorded-observant_imm_ versus Recorded-alone_imm_	− 2.34	127	***0.02***	–	− 0.22	− 0.02
Live_del_ versus Recorded-observant_del_	3.06	127	***0.003***	–	0.06	0.27
Live_del_ versus Recorded-alone_del_	1.86	127	*0.06*	–	− 0.01	0.20
Recorded-observant_del_ versus Recorded-alone_del_	− 1.06	127	*0.29*	–	− 0.18	0.05

ANOVA results for experiment 1, experiment 2 and for the combined dataset. Factors of interest: Group (Neurotypical vs Autistic), Learning Condition (Live vs Recorded-observant vs Recorded-alone) and Time (Immediate vs Delay)

Italic values indicate *p*-values; Bold-italic values indicate *p*-values which reach the threshold for statistical significance of α < .05

*Main effects* Findings show a main effect of time: unsurprisingly, people remembered more things straight after the learning session (mean (sd) = 4.2(0.58) than a week later (Mean = 3.8, *sd* = 0.73; F_(1,44)_ = 56.16, *p* < *0.*001, η^2^ = 0.56, large effect size; Bakeman, 2005). More interestingly, we found a main effect of learning condition (F_(2,43)_ = 3.86, *p* = 0.03, η^2^ = 0.15, medium effect size; Bakeman, 2005). The pairwise comparison revealed a significant learning advantage associated with Live compared to Recorded-alone condition (M_Live_ (sd) = 4.1 (0.09); M_Recorded-alone_ (sd) = 3.9 (0.1); t_(44)_ = 0.19 *p* = 0.008). No other significant difference between learning conditions was found. No main effect was found for group: in other words, autistic people showed an NT-equivalent performance (mean_autistic_ (sd) = 4.01 (0.09); mean_NT_ (sd) = 3.99 (0.1); t_(44)_ = − 0.01 *p* = 0.93).

*Interaction effects* No significant interaction effects were found between the main factors of interest (group, learning conditions and time). However, visualisation of the data (Fig. 1C) revealed a trend specific to the autistic group: while immediate recall showed a similar pattern across conditions between groups, delayed recall dropped specifically for items learned during the Live condition for the autistic group. A 2(group) × 2(time) was therefore run to test the hypothesis that delayed performance was significantly more affected for the autistic group compared to the NT group, specifically for things learned during the Live condition. Results revealed a group*time interaction effect: F_(1,44)_ = 4.88*, p* = 0.03, η^2^ = 0.1): for things learned during Live condition, a week later, autistic people recalled significantly less things compared to NT (mean_autistic_ (sd) = 3.75 (0.18); mean_NT_ (sd) = 3.97 (0.16)).

### Conclusions from experiment 1 & hypotheses for experiment 2

Experiment 1 found that for both NT and autistic people, learning during the Live session was associated with better recall both immediately after the session and one week later. In addition, it was found that the autistic group exhibits a decline in memory for items learned over Live interaction specifically, to a significantly greater extent than what was observed in the NT group. We acknowledge that our sample size (20 autistic adults and 26 neurotypical adults) for experiment 1 is relatively small to conduct a mixed-effects analysis of variance with sufficient power, limiting the strength of our conclusions about the role of social interaction in the learning of autistic individuals. Therefore, based on these results, a follow-up experiment was pre-registered (https://archive.org/details/osf-registrations-5pga3-v1) to confirm two main hypotheses:

1. Participants from both groups will learn more from live calls (Live condition) compared to pre-recorded video calls (Recorded-alone and Recorded-observant condition).
2. There will be an interaction between learning condition, group and time: while neurotypical adults will show a consistent advantage for interactive learning (Live condition) over time, the autistic group will show better immediate learning for material learnt in the Live condition and better long-term learning for materials learned from pre-recorded videos (Recorded-alone and Recorded-observant condition).

## Experiment 2

Experiment 2 consisted of two experimental rounds, when data was collected in parallel by two different researchers (teachers). Each researcher completed the recruitment for the other so that the teacher was always blind to the learner’s diagnosis during data collection. Methods and procedures were identical to Experiment 1. We counterbalanced the items presented in each condition across the two teachers. For teacher 1, items were divided as follows: (1) live condition [*Hulusi, Kiwano, Glaucus, Agogo, Chatelaine*]; (2) recorded-observant condition [*Anhinga, Kalimba, Strigil, Tarsier, Cherimoya*]; (3) recorded-alone condition [*Scotch hands, Axolotl, Rambutan, Porte-joupe, Caxixi*]. For teacher 2, items were divided as follows: (1) live condition [*Anhinga, Cherimoya, Scotch hands, Porte-joupe, Caxixi*]; (2) recorded-observant condition [*Hulusi, Agogo, Chatelaine, Axolotl, Rambutan*]; (3) recorded-alone condition [*Kiwano, Glaucus, Kalimba, Strigil, Tarsier*]. This means that, across the 3 teachers who took part in Experiments 1 and 2, the items are fully counterbalanced between three different experimental conditions.

### Results

#### Sample

86 participants took part in this study (Table 1), split equally between two researchers playing the role of the teacher. Participants were excluded when reporting 3 or less on a 1 (poor) to 5 (excellent) video-call quality scale (N = 4). The final sample included 82 participants (N_autistic_ = 41, male = 17, female = 20, non-binary = 4; N_NT_ = 41, male = 12, female = 27, non-binary = 2).

Autistic participants either received a diagnosed by a clinician (N = 13) or were self-diagnosed (N = 28; see the section below and appendix for further analysis excluding the self-diagnosed participants). The autistic and NT groups did not differ in age (mean_autistic_ (sd) = 27,49 (5,13), mean_NT_ (sd) = 27,63 (4,91), t_(80)_ = 0.13, *p* = 0.89), verbal fluency (Spot the word, mean_autistic_ (sd) = 45.73 (9.83), mean_NT_ (sd) = 44.54 (8.46), t_(80)_ = − 0.59, *p* = 0.56), non-verbal reasoning (MaRs-IB, mean_autistic_ (sd) = 63.08 (18.68), mean_NT_ (sd) = 61.37 (17.26), t_(80)_ = − 0.43, *p* = 0.67) and mentalising test (Animated Triangle, mean_autistic_ (sd) = 9.37 (1.85), mean_NT_ (sd) = 9 (2.20), t_(43)_ = − 0.81, *p* = 0.42). Autistic people scored significantly higher on AQ than NTs (mean_autistic_ (sd) = 28.39, (8.6), mean_NT_ (sd) = 19.98 (6.74); t_(80)_ = − 6.73, *p* < 0.0001).

#### Analysis of variance

**Fig. 3:**
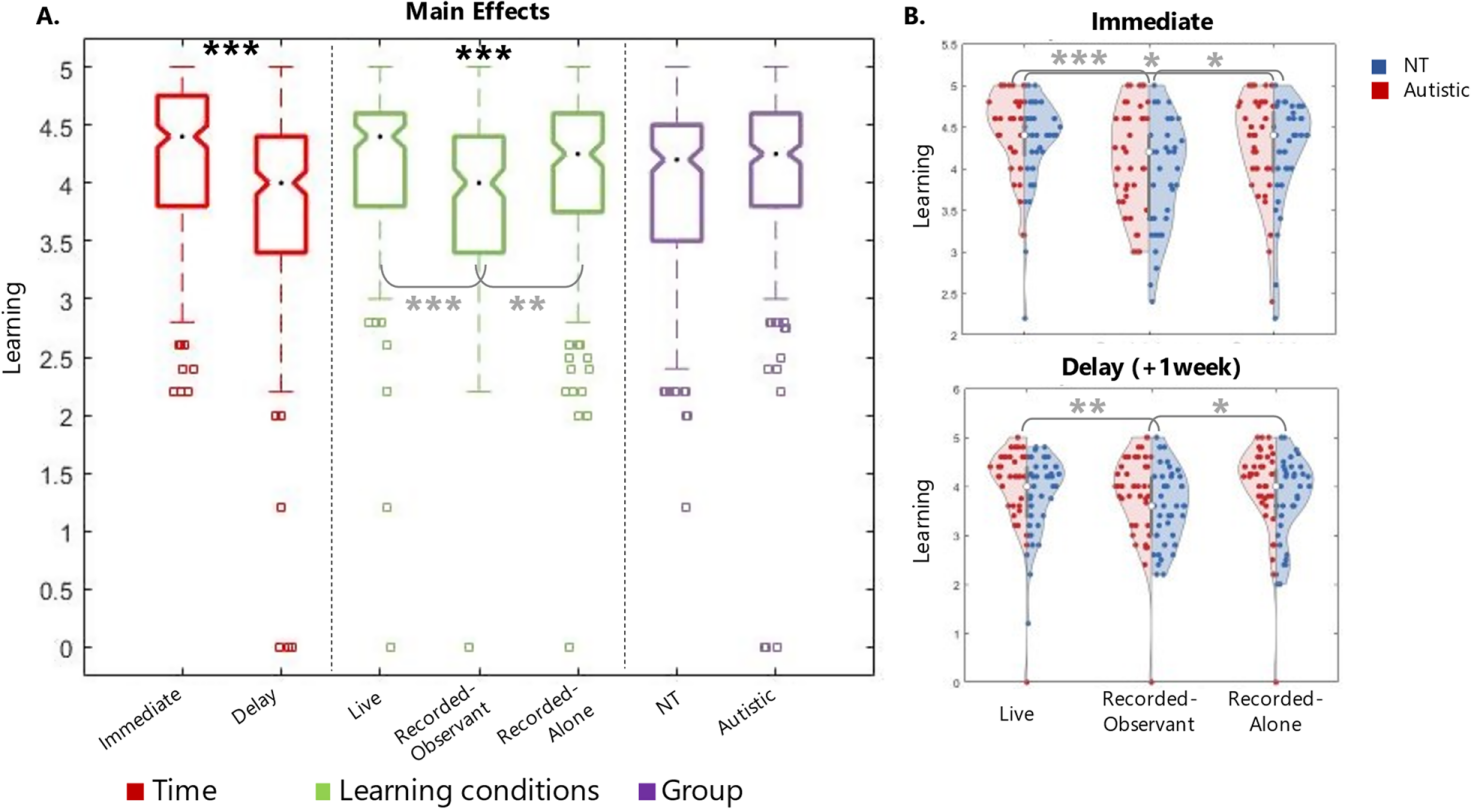
Results for Experiment 2. Results from the dataset of Experiment 2. *p < 0.05; **p < 0.001;***p < 0.0001. **A** Boxplots of the three main factors of interest: Time, Learning condition and Group. **B** Violin plots of learning performance immediately after the learning session (top) and a week later (bottom) for the three learning conditions. Violins are split in half, showing the distribution of NT (blue) and autistic (red) group separately

An analysis of variance (ANOVA) was run to test the difference in learning performance between 2 (groups: AUTISTIC and NT) × 3 (learning conditions: live, recorded-alone and recorded-observant) × 2 (time of learning quiz: immediate and delay). Mean and SD for each condition are reported in Table 2. Results for main and interaction effects are reported in Table 3 and Fig. 3.

*Main effects* Findings show a main effect of time: unsurprisingly, people remembered more things straight after the learning session (mean (sd) = 4.22 (0.6)) than a week later (mean (sd) = 3.83 (0.08); F_(1,80)_ = 38.56, *p* < 0.0001, η^2^ = 0.32, large effect size; Bakeman, 2005). More interestingly, there was a main effect of learning condition (F_(2,80)_ = 13.53, *p* < 0.0001, η^2^ = 0.15, large effect size; Bakeman, 2005). Pairwise comparisons revealed that the Live interaction condition was the one associated with the highest learning. In contrast, Recorded-observant condition was associated with the worst learning: specifically, there was a significant learning advantage associated with Live compared to Recorded-observant condition (M_Live_ (sd) = 4.16 (0.06); M_Recorded-observant_ (sd) = 3.86 (0.07); t_(80)_ = 0.29, *p* < 0.0001), and an advantage approaching significance level compared to Recorded-alone condition (M_Recorded-alone_ (sd) = 4.06 (0.08); t_(80)_ = 0.10, *p* = 0.08). Recorded-observant condition was associated with significantly worse performance than Recorded-alone condition (t_(80)_ = − 0.19, *p = *0.001). No main effect was found for group: in other words, autistic people showed NT-equivalent performance (M_autistic_ (sd) = 4.12 (0.09); M_NT_ (sd) = 3.93 (0.09); t_(44)_ = 0.19, *p* = 0.13).

*Interaction effects* No significant interaction effects were found between the main factors of interest (group, learning conditions and time). Data visualisation showed a similar pattern across groups and times, with the Recorded-observant condition producing the worst learning performance in both groups.

To summarise, this experiment supported hypothesis 1, showing that learning during the Live session was associated with better recall over time for both NT and autistic people. In contrast, hypothesis 2 was not supported: autistic and NT groups showed the same performance pattern over time, with learning over the Recorded-observant condition being associated with the worst performance for both groups.

## Combined analysis

In the previous section, we presented results from experiment 1 (N = 46) and experiment 2 (N = 82). Overall, in both experiments, we found that learning in Live video-call was associated with the best performance for both groups. However, while experiment 1 showed a significant decline in recall over time for the autistic group, specifically for things learned in the Live condition (compared to the NT group), experiment 2 did not confirm this pattern. The fact that hypothesis 2 was not confirmed by experiment 2 could be due to the fact that the effect (decreased live-learning advantage over time for autistic people) is relatively small and more susceptible to individual differences within each sample. Alternatively, the effect observed in experiment 1 may have been a false positive (due to the small sample size). In this section, we present a combined analysis using a mixed-linear effect regression model to better understand the effect of social learning online in autistic and NT participants over time.

### Results

#### Sample

The combined dataset included 128 participants (Table 1, NT N = 67; Autistic N = 61). The autistic group included participants who either received a diagnosed from a clinician (N = 31) or were self-diagnosed (N = 29). All analyses were also run by excluding the self-diagnosed participants in the autistic group. As results did not differ, here we report the whole sample, including the self-diagnosed participants (for results considering only clinically-diagnosed participants refer to Appendix Tables 2, 3 and 4). The autistic and NT group did not differ on age (mean_autistic_ (sd) = 27.58 (6.62), mean_NT_ (sd) = 28.49 (7.27), t_(125)_ = 0.73, *p* = 0.46), verbal fluency (Spot the word, mean_autistic_ (sd) = 46.33, (8.95), mean_NT_ (sd) = 44.61 (7.63), t_(125)_ = 0.64, *p* = 0.24) and non-verbal reasoning (MaRs-IB, mean_autistic_ (sd) = 63.52 (18.50), mean_NT_ = 61.47 (17.02); t_(80)_ = − 0.65, *p* = 0.51) and mentalising test (Animated Triangle, mean_autistic_ (sd) = 9.27 (2.03), mean_NT_ (sd) = 9.08 (2.13), t_(43)_ = − 0.49, *p* = 0.62). Autistic participants scored significantly higher on AQ than NTs (mean_autistic_ (sd) = 29.97 (8.33), mean_NT_ (sd) = 19.67 (6.91), t_(125)_ = − 7.61, *p* < 0.0001). We also confirmed that the teacher was not a significant factor for learning performance (F_(2,126)_ = 0.55, *p* = 0.58), ensuring the dataset could be combined into one despite being collected by different teachers.

**Table 4:** Results from the linear mixed-effects models

	Beta estimate		SE		df		*p-value*		Lower bound		Upper bound	
Model	1	2	1	2	1	2	1	2	1	2	1	2
*Predictors*												
Condition	− 0.07	0.24	0.02	0.14	750	746	***0.003***	*0.089*	− 0.11	− 0.04	− 0.02	0.52
Verbal fluency (Spot the word)^1^	0.02	0.02	0.006	0.006	750	746	***0.002***	***0.0007***	0.01	0.01	0.03	0.03
Non-verbal reasoning (Matrix reasoning item bank, MaRs-IB)^2^	0.01	0.01	0.002	0.002	750	746	***0.0003***	***0.001***	0.004	0.003	0.02	0.01
Mentalising (Animated Triangle)^3^	0.002	− 0.01	0.02	0.02	750	746	*0.91*	*0.57*	− 0.04	− 0.06	0.05	0.03
Autistic Quotient (AQ)^4^	0.001	0.003	0.005	0.14	750	746	*0.78*	*0.53*	− 0.01	− 0.01	0.01	0.01
Enjoyment	–	0.23	–	0.07	–	746	–	***0.0005***	–	0.10	–	0.37
Anxiety	–	0.02	–	0.04	–	746	–	*0.63*	–	− 0.07	–	0.11
Enjoyment*Condition	–	− 0.05	–	0.03	–	746	–	*0.07*	–	− 0.11	–	0.005
Anxiety*Condition	–	− 0.02	–	0.02	–	746	–	*0.33*	–	− 0.07	–	0.02

Outcome of the linear mixed-effects regression models. Model 1: Learning ~ Condition + AQ + Animated Triangle + Spot the word + MaRs-IB + (1 | Participant) + (1|Teacher); Model 2: Learning ~ Condition + AQ + Animated Triangle + Spot the word + MaRs-IB Enjoyment + Enjoyment*Condition + Anxiety + Anxiety*Condition + (1 | Participant) + (1|Teacher)

Italic values indicate *p*-values; Bold-italic values indicate *p*-values which reach the threshold for statistical significance of α < .05

### Mixed-linear regression model

**Fig. 4:**
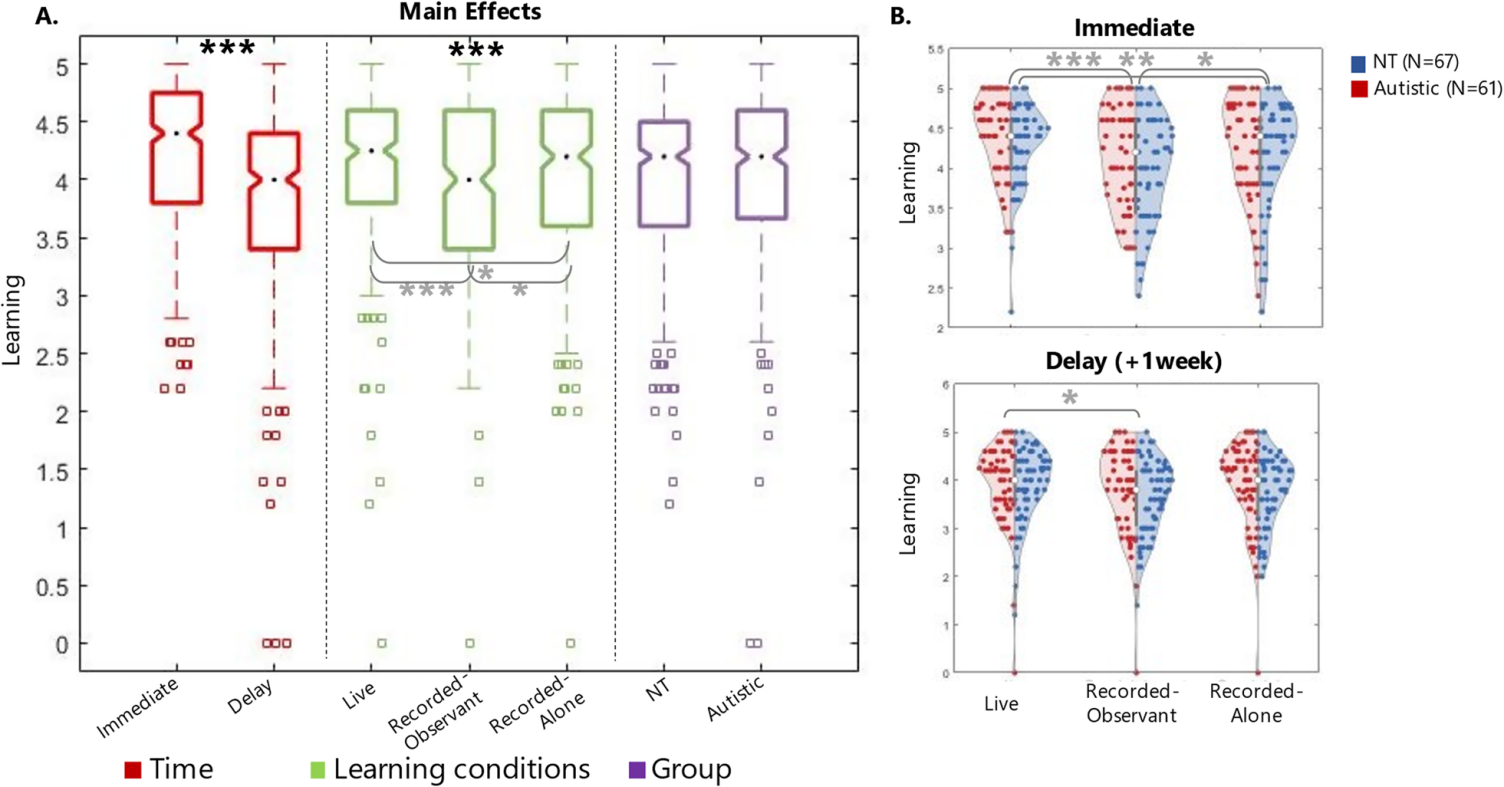
Results for combined analysis (Experiment 1 and 2). **A** Boxplots of the three main factors of interest across the two experiments: Time, Learning condition and Group. **B** Violin plots of learning performance immediately after the learning session (top) and a week later (bottom) for the three learning conditions across the two experiments. Violins are split in half, showing the distribution of NT (blue) and autistic (red) group separately

We use AQ as a continuous measure of autist traits to minimise any confound arising from the fact that our Autistic group included both self-diagnosed and clinically-diagnosed participants. Models were run in Matlab R2020b using the function *fitlme*. Full outcomes for both models are reported in Table 4. Results from this combined analysis are also shown in Fig. 4.

First, we built a model to predict learning performance from the learning condition (Live vs Recorded-observant vs Recorded-alone) along with other variables, including autistic traits (AQ), mentalising (Animated Triangle test), verbal fluency (Spot the word test) and non-verbal reasoning (MaRs-IB) measures, while controlling for variability coming from the teachers and individual participants:

$$\begin{aligned} & Learning \sim Condition + AQ + Animated\;Triangle + Spot\;the\;word + MaRs{ - }IB \\ & + \left( {1|Participant} \right) + \left( {1|Teacher} \right) \\ \end{aligned}$$

Results confirmed ‘Condition’ as a significant predictor of learning performance (beta = − 0.07, *p* = 0.003). In addition, we found that both verbal fluency (Spot the word test, beta = 0.02, *p* = 0.002) and non-verbal reasoning (MaRs-IB, beta = 0.01, *p* = 0.0003) were significant predictors of learning performance. This is not surprising: these measures have been linked to fluid cognition and intelligence [3, 11], which has been robustly associated with learning and academic performance more generally [50]. Regarding measures of autistic traits (AQ) and mentalising (Animated Triangle), we did not find any significant effect, in line with the previous analysis of variance showing no difference between the neurotypical and autistic groups.

Second, in addition to the predictors in Model 1, we included measures of Enjoyment and Anxiety and their interaction with the learning condition.

$$\begin{aligned} & Learning \sim Condition + AQ + Animated\;Triangle + Spot\;the\;word \\ & + MaRs{ - }IB\;Enjoyment + Enjoyment*Condition + Anxiety + Anxiety*Condition \\ & + \left( {1|Participant} \right) + \left( {1|Teacher} \right) \\ \end{aligned}$$

These measures were collected via participant self-report questionnaires after they completed the experiment: Enjoyment measure reflected a score from 1 to 5 for the question ‘*How much did you enjoy learning from the experimenter during [the video-call? / the pre-recorded video?]*’ (1 = Not at all, 5 = Extremely much); Anxiety measure reflected a score from 1 to 5 for the question ‘*How anxious / uncomfortable did you feel when you learned [live from the experimenter? / from the recorded video of another participant? / from the recorded video of the experimenter only?]*’ (1 = Not at all, 5 = Extremely much).

**Fig. 5:**
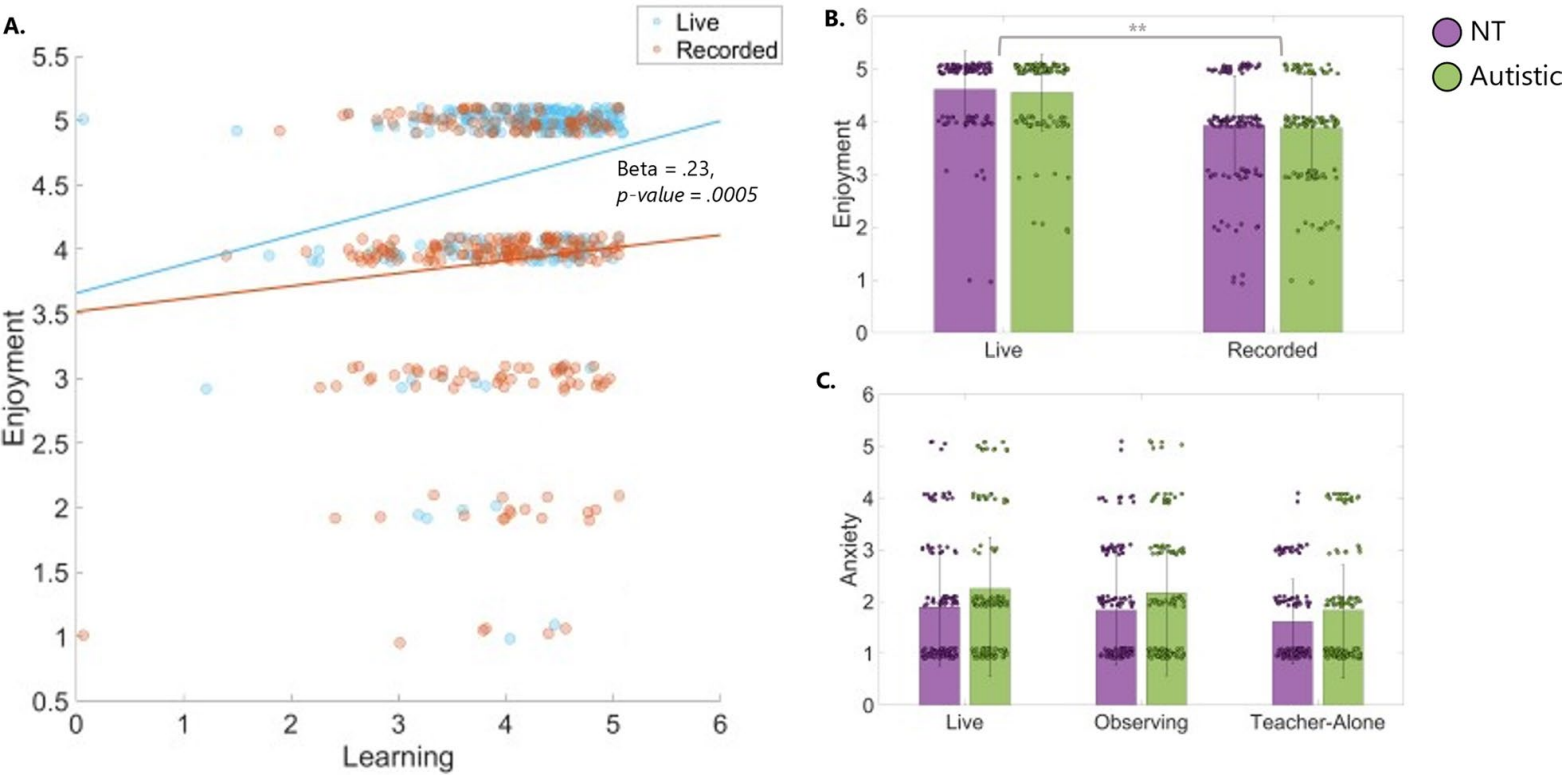
Enjoyment and Anxiety levels across conditions and groups. **A** Scatter plot of learning performance by enjoyment score for both groups, divided by condition (Live and Recorded, note: our questionnaire did not distinguish between the two recorded conditions). **B** Box plots of enjoyment score divided by condition (Live and Recorded, note: our questionnaire did not distinguish between the two recorded conditions) and by group. **C** Box plots of Anxiety score divided by condition and by group. All data plotted here refers to the combined sample (N = 128; NT = 67, Autistic = 61). ** *p* < 0.001

Results show that Enjoyment was a significant predictor of learning for both groups (beta = 0.23, *p* = 0.0005), with people enjoying learning in live interaction (M (sd) = 4.58 (0.71)) significantly more than learning from pre-recorded videos (M (sd) = 3.91 (0.95), t_(128)_ = 7.52, *p* < 0.00001). We also found the Enjoyment*Condition interaction effect approaching significance (beta = − 0.05, *p* = 0.07), with Enjoyment boosting Learning performance slightly more for the Live Condition than Recorded (Fig. 5A), although this is hard to interpret given that we only have Enjoyment scores for Recorded condition overall (i.e. our questionnaire did not make a distinction between Observing and Teacher-Alone condition). Interestingly, compared to Model 1, Condition was no longer a significant predictor of learning performance (beta = − 0.07, *p* = 0.089). Anxiety was not found to be a significant predictor of learning. Neither Enjoyment nor Anxiety scores differ between groups (Fig. 5B and C).

## Discussion

Recently, we found evidence that social interaction boosts learning in neurotypical (NT) adults in online contexts [17]. Here we asked whether the same interactive-learning advantage would replicate in autistic adults. Previous literature on social learning in autism has mainly focused on childhood, with learning tasks including imitation [21], face perception (M. [15, 59] and language [35, 48, 62]. To the best of our knowledge, this is the first large empirical investigation to test interactive-learning of non-social factual knowledge in autistic adults. We tested learning over two blinded experiments (one pre-registered) in 128 adults, equally split into NT and autistic groups, as they were presented with facts about documentary-like content over Zoom in three conditions (Fig. 1): in a live video-call with the teacher (interactive condition), by watching a recorded video of a previous learner-teacher session (recorded-observant condition) and by watching a recorded video of the teacher alone (recorded-alone condition).

We report two main findings: first, overall and across groups, learning in live video-call was significantly greater than learning over pre-recorded videos. This is in line with our pre-registered hypothesis and replicates results from our previous study [17]. Second, the interactive-learning advantage was present in autistic participants too, in accordance with our pre-registered hypothesis 1: in fact, autistic adults not only showed NT-equivalent performance overall but benefitted from learning in live video-call over pre-recorded video as much as NT adults did. Regarding our pre-registered hypothesis 2, the present data do not support it: we found no difference between groups in learning-advantage over time. In other words, the social learning advantage did not fade over time for autistic people and was similar when tested immediately after the session and one week later. The mixed-linear regression model run on the whole sample over the two experiments (combined analysis) confirmed the similar learning pattern between groups over time. We believe the greater decline in recall for materials learned in the live condition observed in autistic participants in experiment 1 may have been a false positive, consistent with the small sample size of our first experiment.

Several mechanisms including attention and mutual-understanding may support learning in a contingent learner-teacher interaction [16]. Our investigation does not allow us to disentangle these different mechanisms nor to interpret NT and autistic group performance with reference to specific cognitive processes. Past studies suggest that candidate mechanisms related to the interactive-learning benefit—including attention, social motivation and reward, mentalising and arousal—have been found to various degrees to be atypical in autistic individuals [2, 12, 25, 28, 32, 38, 59, 60]. This would predict poorer learning in autistic people in social contexts. However, our results robustly contradict this prediction. Suppose the cognitive processes implicated in interactive social learning are also those typically disrupted in autistic people. Why do we still find that autistic participants learned as well as NT in our study? We identified two possible explanations: (i) autistic people showed NT-equivalent performance, but at a greater cost (compensatory hypothesis), (ii) while autistic people may struggle to learn *about* ‘the social’, they benefit from learning via ‘the social’ as much as NT do (about-the-social versus via-the-social hypothesis).

First, it may be that equivalent learning between groups comes at a greater cost for autistic people (compensatory hypothesis). Both hyper- or hypo-arousal during social information processing have been associated with autistic people [65]. Autistic people also show abnormalities in executive function, sensory processing and emotional regulation [22, 37, 54]. This may result in the social environment being cognitively demanding for autistic people and would predict that they may show discomfort and/or less enjoyment during the task, despite overall NT-equivalent performance. Our results, however, reject this interpretation. The autistic group *enjoyed* the experiment as much as the NT group and significantly more when learning in the interactive condition than in the less-interactive ones (pre-recorded videos). Crucially, anxiety levels during the task also did not significantly differ between the two groups nor across learning conditions. This data is in line with previous work suggesting that autistic people do not lack social motivation [34, 44, 66]. Equivalent preference for the live condition between groups clearly argue for social motivation being intact in our sample of autistic participants. However, social motivation alone cannot explain the interactive learning advantage reported here in the autistic group. In fact, while someone may enjoy learning in a live interaction more than from recorded videos, this does not always necessarily translate into better performance. Also, even if that was the case, we would still expect some group differences—which we do *not* find –, given that our autistic sample showed significant differences on social communication and attention compared to neurotypical adults (as measured by the AQ, [4].

Instead, we suggest that this data supports a distinction between learning *about-the-social versus via-the-social*. Most of the past literature has failed to disentangle these two phenomena experimentally: previous experimental work looked at how autistic people either learn *about* the social information, for example facial emotions. Studies of imitation have looked at how autistic people use *implicit social signals* to decide when to imitate and learn [23]. In contrast, the present work disentangled *the means* through which *explicit* learning occurs from *the object* of learning: this may have allowed autistic people to benefit from the same interactive mechanisms supporting learning in NT. While the present data do not allow us to identify the specific process, or set of processes, responsible for supporting learning in interactive contexts, it robustly showed how crucial it is to separate the medium through which we learn from the content of learning and how contingent social interaction in online context can act as a catalyst for learning across a variety of people, inclusive of autistic groups. The ‘*about* versus via the social’ hypothesis seems to provide a comprehensive explanation for the social learning advantage in autistic people reported here, and provides a useful framework to test different hypotheses in future experiments.A possible confound for the learning advantage we observed during live sessions is that during the interaction, the learner can ask questions, effectively resulting in repetitions of the learning content that are absent in non-interactive learning. However, we do not believe the interactive social learning advantage is due to a disparity in content exposure across conditions. Instead, we propose that the *‘potential to’* interact—typical of contingent sessions—triggers a different ‘intentional stance’ [18] or, differently put, a unique mental state [31] that elicits specific cognitive and physiological processes that are not active during non-contingent sessions (e.g. watching a video). This qualitatively different state may support learning through mechanisms of (shared) attention and common ground [6, 19]. In line with this proposition, and although we did not measure the number of interactions here, it is noteworthy that in the recorded conditions specifically, learning was significantly better when the teacher was alone compared to observing the recorded video of the teacher interacting with another learner (who asked questions etc.). This is consistent with our previous study [17], which used the same material and a similar paradigm but adopted a yoked design where the live session of participant 1 acted as the recorded session for participant 2. Therefore, the exact same interactions appeared in both conditions, yet learning in live contingent interaction was significantly better. This evidence suggests two main conclusions: first, an abundance of social signals may be beneficial only when *we are part of* the interaction—as a way to improve communication, but not when we observe one, when additional signals may just be a cognitive cost; and 2) what drives the learning advantage is something unique to contingent social exchange: being part of social interaction may ‘switch on’ a series of cognitive, computational, physiological and neural processes [18, 26] that may support learning *with* others in ways that are not possible when we simply learn *from* others [16]. Another possibility however is that live sessions allow interactions (e.g. questions) tailored to the learner’s needs, which may be different across different learners. Future studies should directly test this hypothesis.

This work has the strength of studying a novel question in a large sample, with findings robustly replicated over two separate experiments and three different blinded teachers. Results are ground-breaking in that they demonstrate, for the first time, that autistic adults *benefit* and *enjoy* learning via live-interaction more than from recorded videos. Some considerations, however, must be made to place these findings within the appropriate panorama. About 50% of the autistic participants who took part in this study were self-diagnosed. Although results did not differ when these participants were excluded, conclusions may not extend to the entire autism population. Moreover, recruiting participants over online platforms may attract people who are generally keen to participate in interactive social experiments while leaving out those less likely to engage in social interaction (either because they struggle more or because they enjoy it less). Future work should investigate this question in a sample recruited via diverse sources (e.g. schools). In addition, future studies should take consider individual preferences for learning: while we counterbalanced items across conditions, it may have still been the case that individual differences may have made certain items more interesting and possibly easier to learn.

Our results predict that the interactive-learning advantage would also apply to in-person contexts. The present study—despite being online—resembled more the typical in-person situation: participants did not engage with any of the defining characteristics of a typical e-learning experience, e.g. pausing, repeating, forwarding etc. We acknowledge that other aspects specific to in-person interaction, e.g. arousal due to physical proximity [41, 43] and sensory differences between online and offline contexts, may play a role in learner-teacher interaction, and even be reflected in different learning preferences in autistic and non-autistic people. Online versus face-to-face contexts should be directly tested. In addition, our design studies social learning in dyadic exchange. We believe this provides the smallest unit of real-world social exchange. Understanding the details of naturalistic one-to-one interaction can be useful in building models that can later be tested in larger real-world social contexts. For example, in real-world school settings, larger group dynamics involve many social (and non-social) signals, which may modulate learning differently depending on intra-individual and inter-individual processes. We showed here that social interaction supports learning even in neurodiverse groups typically associated with poor social cognition. This means that social interaction is an effective *means* through which people learn. We do not know yet how the interactive social learning advantage observed in dyadic interaction transfers to large group situations. Future studies could further investigate whether and how other (social) factors modulate this effect in different social contexts.

In conclusion, we showed that autistic people benefit and enjoy learning in interactive contexts as much as NT people do while not reporting being more anxious in any of our interactive social conditions. The present work has implications for classic cognitive models of social learning, arguing for a distinct separation of the context through which learning occurs from the content of learning. Designing experiments that separate these two factors is essential to better understand the underlying cognitive mechanisms supporting interactive social learning in both neurotypical and neuro-diverse populations. This would help identify specific dysfunctions of social cognition without making assumptions about a certain condition in relation to one factor (e.g. via-the-social) based solely on the other (e.g. about-the-social). Practical implications for pedagogy include re-thinking how we deal with education in autism.

### Supplementary Information

**Additional file 1: Table 1.** Full set of items and quiz for the learning task. **Table 2.** Demographic information for NTs and clinically-diagnosed autistic participants. **Table 3.** Sample size (N), Means and SDs for all conditions for NTs and clinically-diagnosed autistic participants. **Table 4.** Results for clinically-diagnosed autistic participants only.

## Data Availability

Experiment 1 was not preregistered; the preregistration for Experiment 2 can be accessed at https://osf.io/5pga3. Deidentified data for both experiments are posted at https://osf.io/5az7g/?view_only=27d98b16261f47a6a9e1c9de6c053660. The materials used in these studies are available in Supplementary Material, uploaded as a separate file with this submission.

## References

[1] American Psychiatric Association. Diagnostic and statistical manual of mental disorders, 5th ed., text rev.; 2022. 10.1176/appi.books.9780890425787

[2] Abell F, Happé F, Frith U (2000). Do triangles play tricks? Attribution of mental states to animated shapes in normal and abnormal development. Cogn Dev.

[3] Baddeley A, Emslie H, Nimmo-Smith I (1993). The Spot-the-Word test: a robust estimate of verbal intelligence based on lexical decision. Br J Clin Psychol.

[4] Baron-Cohen S, Wheelwright S, Skinner R, Martin J, Clubley E (2001). The autism-spectrum quotient (AQ): evidence from Asperger syndrome/high-functioning au-tism, males and females, scientists and mathematicians [erratum appears in J Autism Dev Dis-ord 2001 Dec;31(6):603]. J Autism Dev Disord.

[5] Bernier R, Dawson G, Webb S, Murias M (2007). EEG mu rhythm and imitation impairments in individuals with autism spectrum disorder. Brain Cogn.

[6] Bohn M, Tessler MH, Frank M (2019) Integrating common ground and informativeness in pragmatic word learning. 10.31234/osf.io/cbx46

[7] Bottini S. Social reward processing in individuals with autism spectrum disorder: a systematic review of the social motivation hypothesis. In: Research in autism spectrum disorders, vol. 45, Elsevier Ltd; 2018. pp. 9–26. 10.1016/j.rasd.2017.10.001

[8] Buch AM, Vértes PE, Seidlitz J, Kim SH, Grosenick L, Liston C (2023). Molecular and network-level mechanisms explaining individual differences in autism spectrum disorder. Nat Neurosci.

[9] Čeponiene R, Lepistö T, Shestakova A, Vanhala R, Alku P, Näätänen R, Yaguchi K (2003). Speech-sound-selective auditory impairment in children with autism: they can perceive but do not attend. Proc Natl Acad Sci USA.

[10] Chevallier C, Kohls G, Troiani V, Brodkin ES, Schultz RT (2012). The social motivation theory. Trends Cogn Sci.

[11] Chierchia G, Fuhrmann D, Knoll LJ, Pi-Sunyer BP, Sakhardande AL, Blakemore SJ (2019). The matrix reasoning item bank (MaRs-IB): novel, open-access abstract reasoning items for adolescents and adults. R Soc Open Sci.

[12] Clements CC, Zoltowski AR, Yankowitz LD, Yerys BE, Schultz RT, Herrington JD (2018). Evaluation of the social motivation hypothesis of autism a systematic review and meta-analysis. JAMA Psychiat.

[13] Corbett BA, Swain DM, Newsom C, Wang L, Song Y, Edgerton D (2014). Biobehavioral profiles of arousal and social motivation in autism spectrum disorders. J Child Psychol Psychiatry.

[14] Dawson G, Meltzoff AN, Osterling J, Rinaldi J, Brown E (1998). Children with autism fail to orient to naturally occurring social stimuli. J Autism Dev Disord.

[15] Dawson M, Mottron L, Gernsbacher MA (2007). Learning in autism. Learn Mem Compr Ref.

[16] De Felice S, Hamilton AFC, Ponari M, Vigliocco G (2022). Learning from others is good, with others is better: the role of social interaction in human acquisition of new knowledge. Philos Trans R Soc B Biol Sci.

[17] De Felice S, Vigliocco G, Hamilton AFC (2021). Social interaction is a catalyst for adult human learning in online contexts. Curr Biol.

[18] Devaine M, Hollard G, Daunizeau J (2014). The social Bayesian brain: Does mentalizing make a difference when we learn?. PLoS Comput Biol.

[19] Dideriksen C, Christiansen MH, Kristian T, Dingemanse M, Fusaroli R (2020) Building common ground: quantifying the interplay of mechanisms that promote understanding in conversations Building common ground: quantifying the interplay of mechanisms that promote understanding in conversations, pp 1–156.

[20] Dubey I, Ropar D, Hamilton AF (2015). Measuring the value of social engagement in adults with and without autism. Mol Autism.

[21] Edwards LA (2014). A meta-analysis of imitation abilities in individuals with autism spectrum disorders. Autism Res.

[22] Fernandez-Prieto M, Moreira C, Cruz S, Campos V, Martínez-Regueiro R, Taboada M, Carracedo A, Sampaio A (2021). Executive functioning: a mediator between sensory processing and behaviour in autism spectrum disorder. J Autism Dev Disord.

[23] Forbes PAG, Suddell SF, Farmer H, Logeswaran Y, Hamilton AFDC (2019). The way others move can influence what we choose. Q J Exp Psychol.

[24] Fitch WT, Huber L, Bugnyar T (2010). Social cognition and the evolution of language: constructing cognitive phylogenies. Neuron.

[25] Frazier TW, Strauss M, Klingemier EW, Zetzer EE, Hardan AY, Eng C, Youngstrom EA (2017) A meta-analysis of gaze differences to social and nonsocial information between individuals with and without autism, vol. 56. www.jaacap.org 10.1016/j.jaac.2017.05.005 PMC5578719 28647006

[26] Friston K, Frith C (2015). A duet for one. Conscious Cogn.

[27] Hamilton AFdeC (2013). Reflecting on the mirror neuron system in autism: a systematic review of current theories. Dev Cogn Neurosci.

[28] Hamilton AFdeC, Lind F (2016). Audience effects: what can they tell us about social neuroscience, theory of mind and autism?. Cult Brain.

[29] Happé F, Frith U (2005). Autism spectrum disorder. Curr Biol.

[30] Happé F, Frith U (2014). Annual research review: towards a developmental neuroscience of atypical social cognition. J Child Psychol Psychiatry.

[31] Hasson U, Frith CD (2016). Mirroring and beyond: coupled dynamics as a generalized framework for modelling social interactions. Philos Trans R Soc B Biol Sci.

[32] Hill EL (2004). Executive dysfunction in autism. Trends Cogn Sci.

[33] Hirschfeld L, Bartmess E, White S, Frith U (2007) Can autistic children predict behavior by social stereotypes? In: Current biology, vol. 17, no. 12. Cell Press. 10.1016/j.cub.2007.04.051 10.1016/j.cub.2007.04.051 17580071

[34] Jaswal VK, Akhtar N (2019). Being versus appearing socially uninterested: challenging assumptions about social motivation in autism. Behav Brain Sci.

[35] Kasari C, Paparella T, Freeman S, Jahromi LB (2008). Language outcome in autism: randomized comparison of joint attention and play interventions. J Consult Clin Psychol.

[36] Kenny L, Hattersley C, Molins B, Buckley C, Povey C, Pellicano E (2016). Which terms should be used to describe autism? Perspectives from the UK autism community. Autism.

[37] Kilroy E, Aziz-Zadeh L, Cermak S (2019). Ayres theories of autism and sensory integration revisited: what contemporary neuroscience has to say. Brain Sci.

[38] Klin A (1991). Young autistic children’s listening preferences in regard to speech: a possible characterization of the symptom of social withdrawal. J Autism Dev Disord.

[39] Leekam S (2016). Social cognitive impairment and autism: What are we trying to explain?. Philos Trans R Soc B Biol Sci.

[40] Livingston LA, Shah P, White SJ, Happé F (2021). Further developing the Frith-Happé animations: A quicker, more objective, and web-based test of theory of mind for autistic and neurotypical adults. Autism Res.

[41] Lougheed JP, Koval P, Hollenstein T (2016). Sharing the burden: The interpersonal regulation of emotional arousal in mother-daughter dyads. Emotion.

[42] Marsh L, Pearson A, Ropar D, Hamilton A (2013). Children with autism do not overimitate. Curr Biol.

[43] McBride G, King MG, James JW (1965). social proximity effects on galvanic skin responses in adult humans. J Psychol.

[44] McNaughton KA, Kirby LA, Warnell KR, Alkire D, Merchant JS, Moraczewski D, Yarger HA, Thurm A, Redcay E (2023). Social-interactive reward elicits similar neural response in autism and typical development and predicts future social experiences. Dev Cogn Neurosci.

[45] Monk R, Whitehouse AJO, Waddington H (2022). The use of language in autism research. Trends Neurosci.

[46] Mundy P (1995). Joint attention and social-emotional approach behavior in children with autism. Dev Psychopathol.

[47] Mundy P, Newell L (2007). Attention, joint attention & social cognition. Soc Cogn.

[48] Norbury CF, Griffiths H, Nation K (2010). Sound before meaning: Word learning in autistic disorders. Neuropsychologia.

[49] Pearson J (2004) Autism and learning disability, pp. 50–60. 10.1177/1362361304042718 10.1177/1362361304042718 15165430

[50] Primi R, Ferrão ME, Almeida LS (2010). Fluid intelligence as a predictor of learning: a longitudinal multilevel approach applied to math. Learn Individ Differ.

[51] Riby DM, Hancock PJB (2008). Viewing it differently: social scene perception in Williams syndrome and Autism. Neuropsychologia.

[52] Roos EM, McDuffie AS, Weismer SE, Gernsbacher MA (2008). A comparison of contexts for assessing joint attention in toddlers on the autism spectrum. Autism.

[53] Sasson NJ, Turner-Brown LM, Holtzclaw TN, Lam KSL, Bodfish JW (2008). Children with autism demonstrate circumscribed attention during passive viewing of complex social and nonsocial picture arrays. Autism Res.

[54] Semrud-Clikeman M, Walkowiak J, Wilkinson A, Butcher B (2010). Executive functioning in children with asperger syndrome, ADHD-combined type, ADHD-predominately inattentive type, and controls. J Autism Dev Disord.

[55] Seyfarth RM, Cheney DL (2014). The evolution of language from social cognition. Curr Opin Neurobiol.

[56] Spengler S, Bird G, Brass M (2010). Hyperimitation of actions is related to reduced understanding of others’ minds in autism spectrum conditions. Biol Psychiatry.

[57] Todorova GK, Hatton REMB, Pollick FE (2019). Biological motion perception in autism spectrum disorder: a meta-analysis. Mol Autism.

[58] Uljarevic M, Hamilton A (2013). Recognition of emotions in autism: a formal meta-analysis. J Autism Dev Disord.

[59] Webb SJ, Neuhaus E, Faja S (2017). Face perception and learning in autism spectrum disorders. Q J Exp Psychol.

[60] White SJ (2013). The triple i hypothesis: taking another('s) perspective on executive dysfunction in autism. J Autism Dev Disord.

[61] White SJ, Coniston D, Rogers R, Frith U (2011). Developing the Frith-Happé animations: a quick and objective test of theory of mind for adults with autism. Autism Res.

[62] Whitehouse AJO, Barry JG, Bishop DJM (2007). The broader language phenotype of autism: a comparison with specific language impairment. J Child Psychol Psychiatry.

[63] Williams JHG, Whiten A, Suddendorf T, Perrett DI (2001). Imitation, mirror neurons and autism. Neurosci Biobehav Rev.

[64] Xie H, Moraczewski D, Mcnaughton KA, Warnell KR, Alkire D, Merchant JS, Kirby, LA, Yarger HA, Redcay E (pre-print). Social reward network connectivity differs between autistic and neurotypical youth during social interaction. 10.1101/2023.06.05.543807

[65] Yi L, Wang Q, Song C, Han ZR (2022). Hypo- or hyperarousal? The mechanisms underlying social information processing in autism. Child Dev Perspect.

[66] Yurkovic-Harding J, Lisandrelli G, Shaffer RC, Dominick KC, Pedapati EV, Erickson CA, Yu C, Kennedy DP (2022). Children with ASD establish joint attention during free-flowing toy play without face looks. Curr Biol.

