# Supplementary material for: Autistic adults benefit from and enjoy learning via social interaction as much as neurotypical adults do
Source: Mol Autism. 2023 Sep 6;14:33. doi: 10.1186/s13229-023-00561-6 (PMC10481576; doi:10.1186/s13229-023-00561-6)
Supplement: Supplementary file 1 — Additional file 1: Table 1. Full set of items and quiz for the learning task. Table 2. Demographic information for NTs and clinically-diagnosed autistic participants. Table 3. Sample size (N), Means and SDs for all conditions for NTs and clinically-diagnosed autistic participants. Table 4. Results for clinically-diagnosed autistic participants only. [file 13229_2023_561_MOESM1_ESM.docx]

# Table 1 – Full set of items and quiz for the learning task.

| Item and learning material | Multiple-choice quiz |
| --- | --- |
| 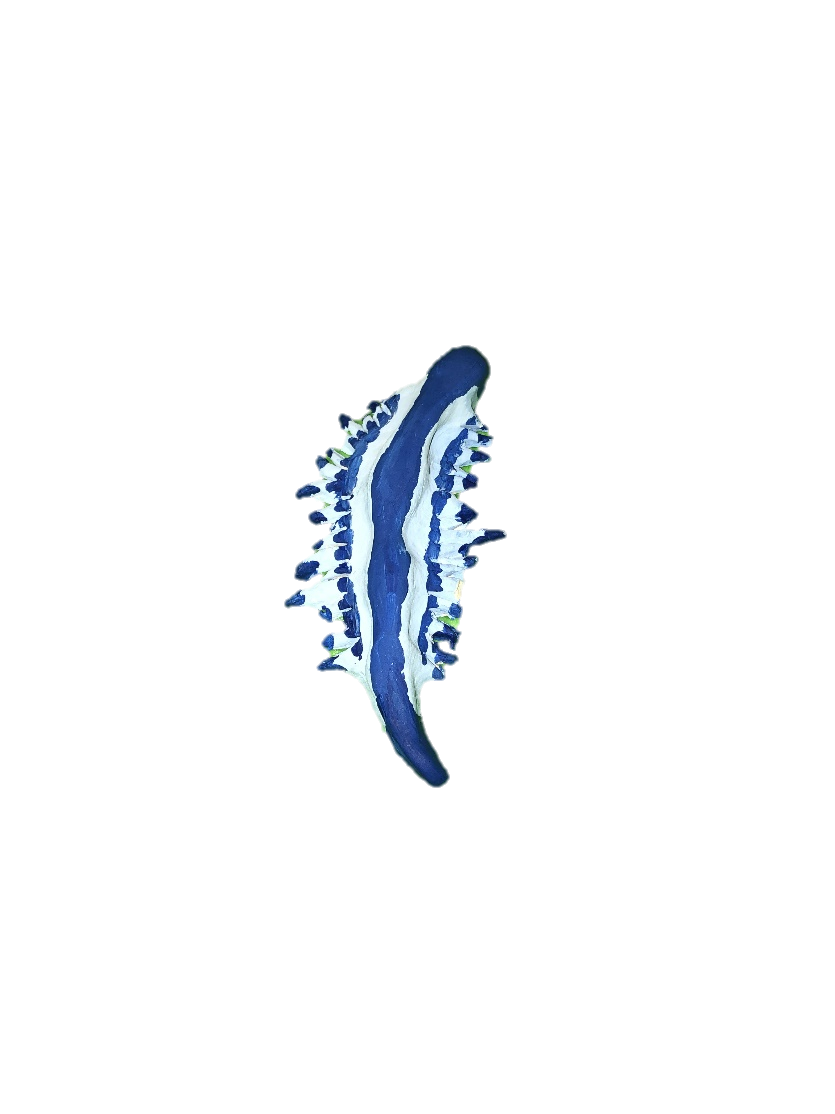1. GLAUCUS  Glaucus is a mollusc. It lives in the ocean, especially around India and Indonesia. It is known as ‘the blue dragon’ because of its colour and ray-shape tentacles. It feeds on jellyfish. However the jellyfish is not digested completely: the most poisoning cells are stored in the tips of the Glaucus tongue (in-between teeth) and used as a self-defence tool. It has a gas-filled sac in its stomach which acts as a balloon allowing it to float on the surface of the water: they don't swim but are carried along by the winds and ocean currents. | 1. What species is it?    1. Fish    2. Mollusc    3. Mammal 2. What is its habitat?    1. Ocean    2. Lake    3. Tropical forest 3. How is it also known as and why? 4. "Blue dragon" because of its colour and shape 5. "Cold fire" because of the blue ray-shape fingers 6. "Sea snake" because of the long tail 7. What does it eat? 8. Fish 9. Leaves 10. Jellyfish 11. How can it float? 12. Thanks to its ray-shape fingers 13. It doesn't: it lives deep on the bottom of the ocean 14. Via a gas-filled sac in its stomach |
| 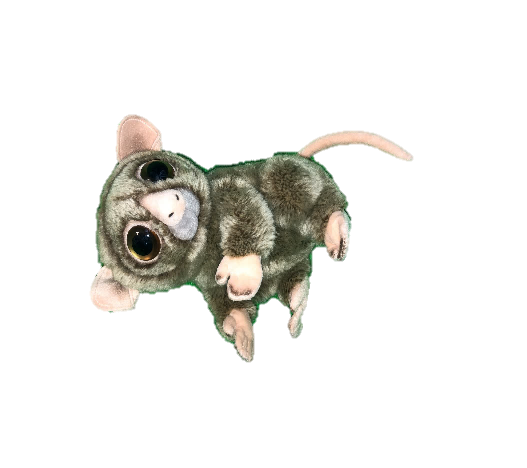2. TARSIER  Tarsier is a mammal. Originally from south-east Asia, it lives in the jungle. Its name comes from its very long tarsal (ankle) bone. The elongated legs allow it to jump from tree to tree hunting prey (it can jump up to 40 meters). It is totally carnivorous and eats mainly insects. It has special eyes: these are bigger and heavier than its brain. Because its eyes are so huge, it cannot move them and has to rotate its head to look around. The head can rotate 180 degrees. Such big eyes allow for very good night vision. | 1. What species is it?    1. Bird    2. Amphibian    3. Mammal 2. What is its habitat?    1. Jungle    2. Mountain    3. Fresh water 3. Where does the name come from?    1. From its long fingers    2. From its long ankle bone    3. From the region it lives in 4. What does it eat?    1. Only carnivorous, mostly insects    2. Only herbivorous, mainly bananas    3. Omnivorous, mainly insects 5. What's special about its eyes?    1. They act like magnifying glasses to detect extremely small insects    2. They are bigger and heavier than its brain and can see in the dark    3. They are bright yellow to be able to see in the dark |
| 3. KIWANO 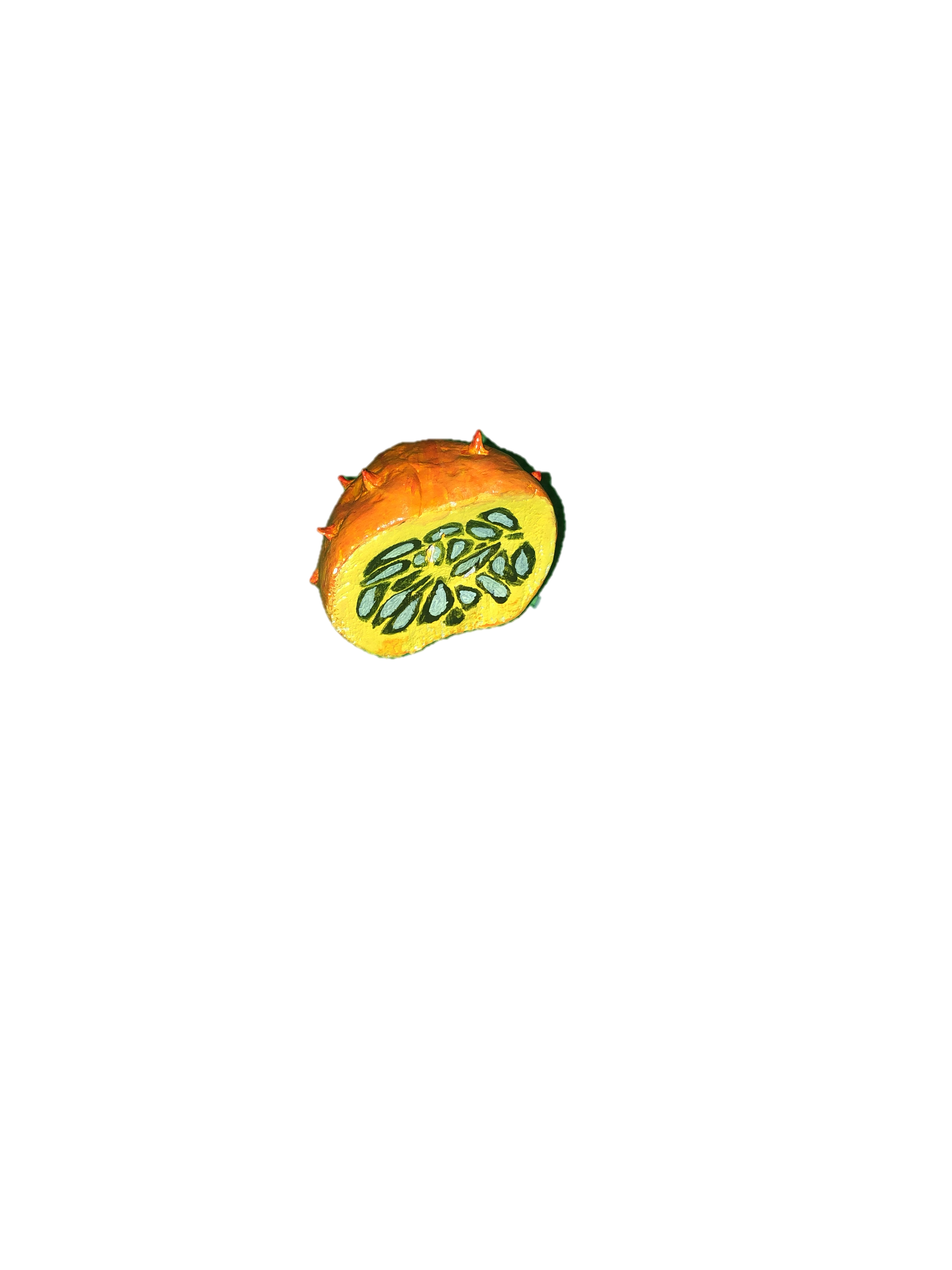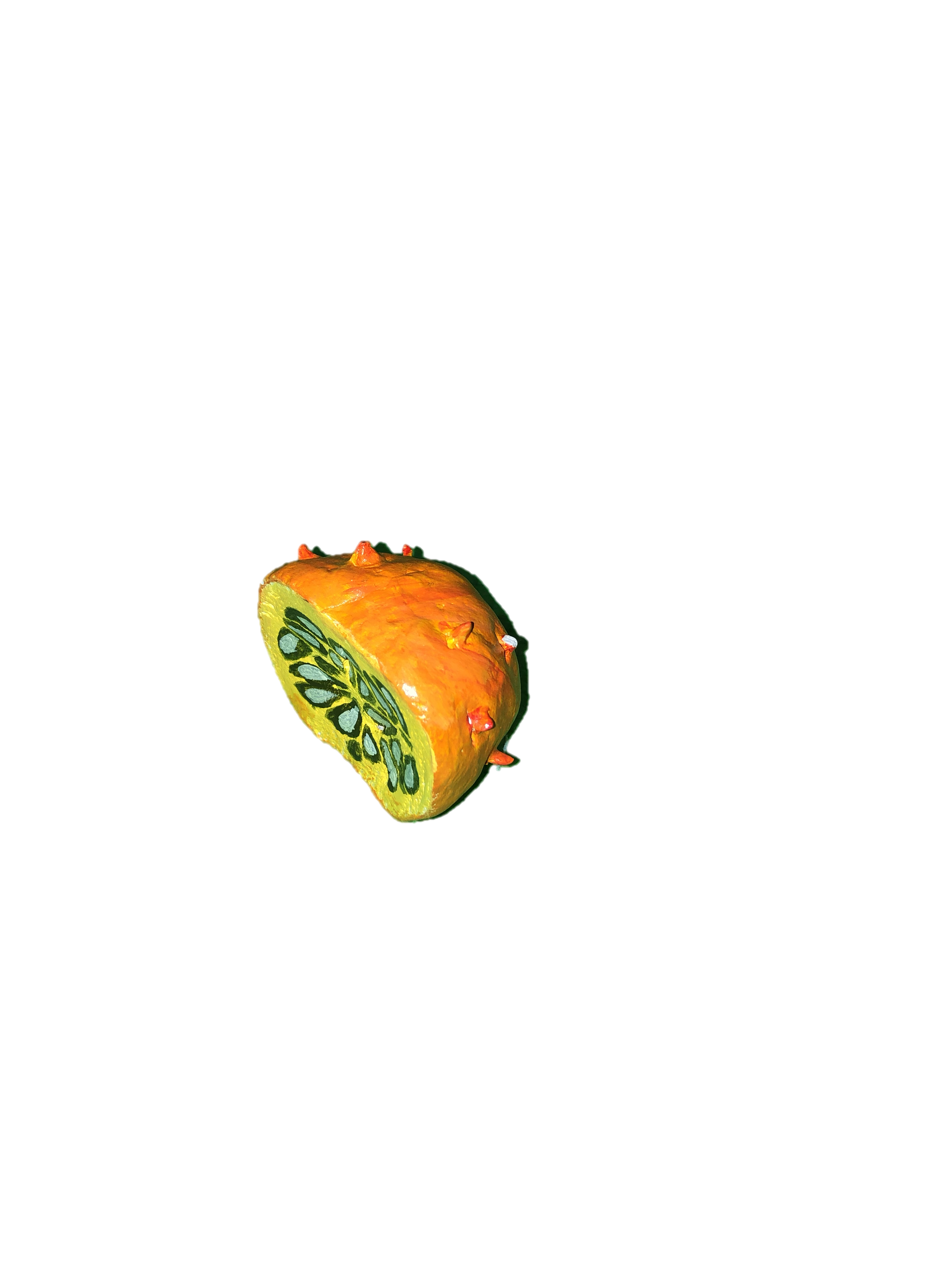 Kiwano is a fruit native of Sub-Saharan Africa and needs a warm climate to grow. It is also known as the 'horned melon' because of its melon-like colour and the fact that it presents some horns/spikes on its skin. All its parts, including seeds, flesh, and peel, are edible. Its flavour is a combination of banana, cucumber, and lime: so quite fresh and with a gelatine-like texture. It is a good source of vitamins and liquids, and represents one of the few sources of water in Africa deserts during the dry season. It grows for half of the year from January until July. | 1. Where is it originally from?    1. South-East Asia    2. Sub-Saharan Africa    3. Northern Africa 2. How is it also known and why? 3. Orange melon because of its colour 4. Spiked peach because of its skin 5. Horned melon because of its skin 6. What can/cannot be eaten? 7. It is all edible 8. The peel cannot be eaten 9. The seeds are poisonous 10. What does it taste like? 11. Strawberries, lemon, and melon 12. Banana, cucumber, and lime 13. Spinach, peas and lemon 14. In which season(s) does it grow? 15. Only during summer 16. From July to November 17. From January to July |
| 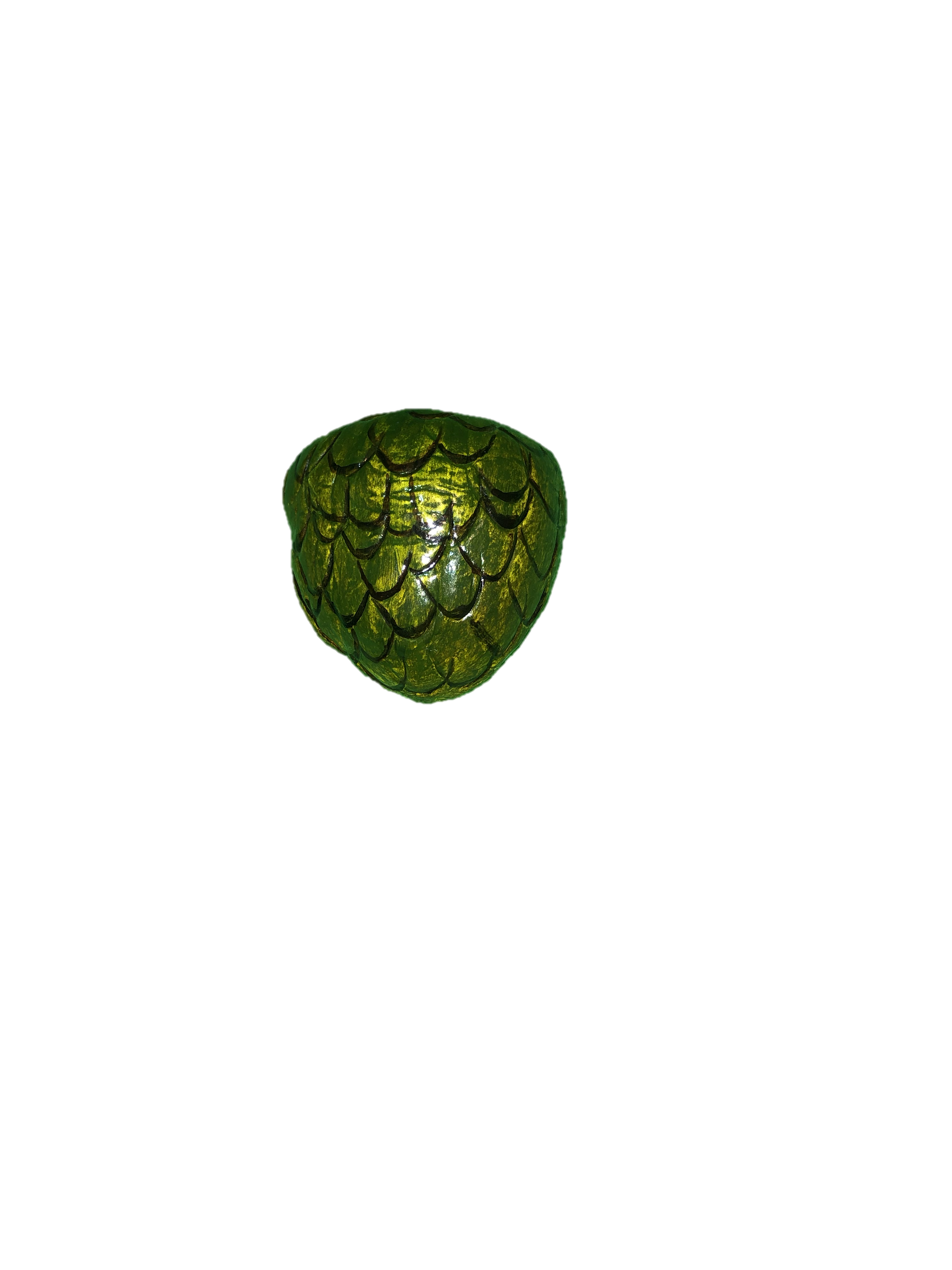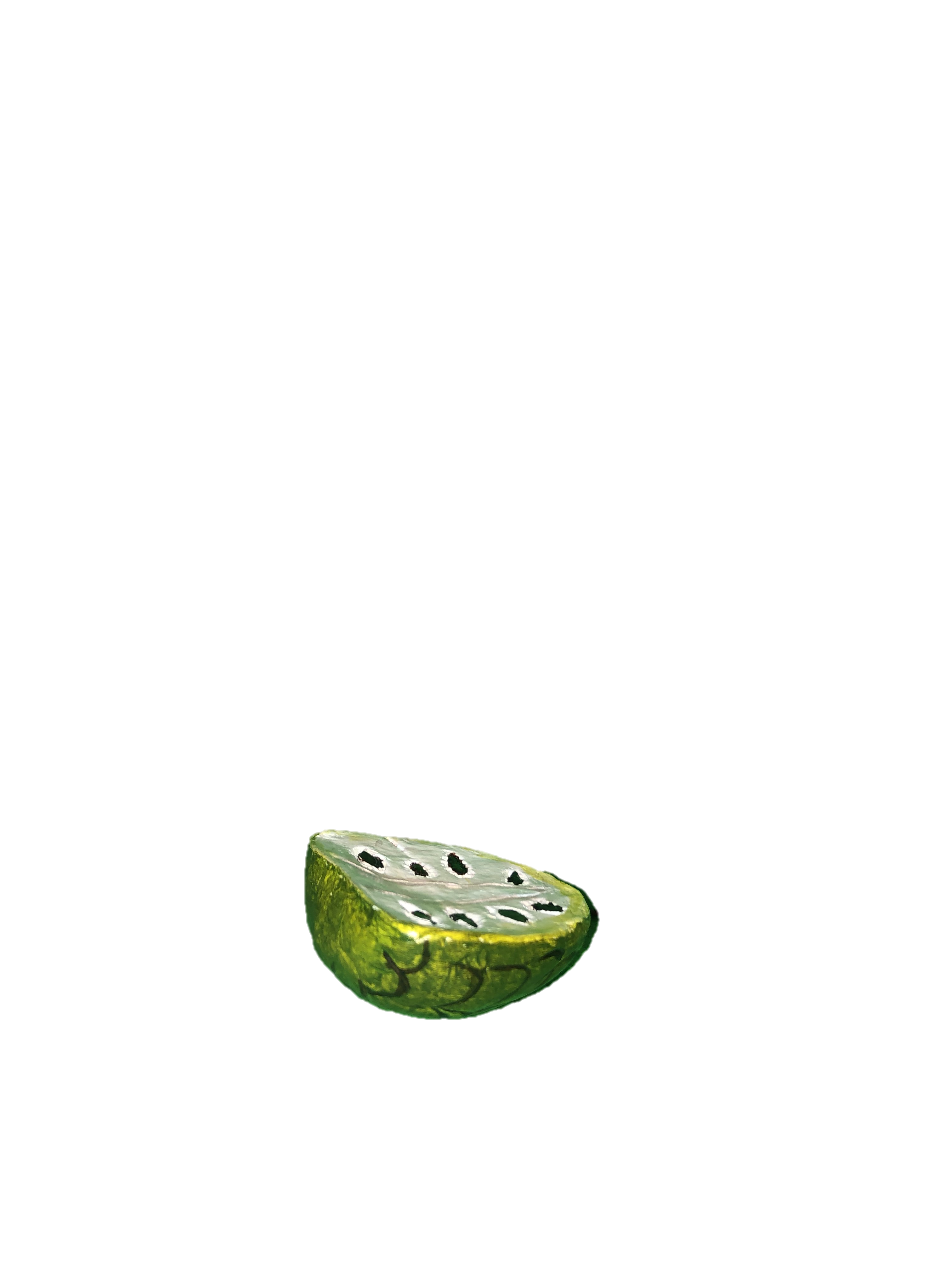4. CHERIMOYA  Cherimoya is a fruit native of South America. Its name means “cold seeds”, because it grows at high altitude where the climate is cold. The whitish flesh is edible, however its seeds are poisonous especially if eaten in big quantities (these are usually used to make pesticides). The flesh tastes like a blend of banana, pineapple, peach, and strawberry. It is very nutrient and has high calories with lots of good vitamins. It ripens from autumn until spring. When ripe the skin is green, but it turns brown when goes rotten. | 1. Where is it originally from? 2. South America 3. North America 4. South-east Asia 5. What does its name mean? 6. "Spring taste" 7. "Rough skin" 8. "Cold seeds" 9. What can/cannot be eaten? 10. It is all edible 11. The seeds are poisonous 12. The peel cannot be eaten 13. What does it taste like? 14. Sweet potatos 15. Grapes, lemon, apple and pear 16. Banana, pineapple, peach and strawberry 17. In which season(s) does it grow? 18. Autumn to spring 19. Winter to summer 20. Summer only |
| 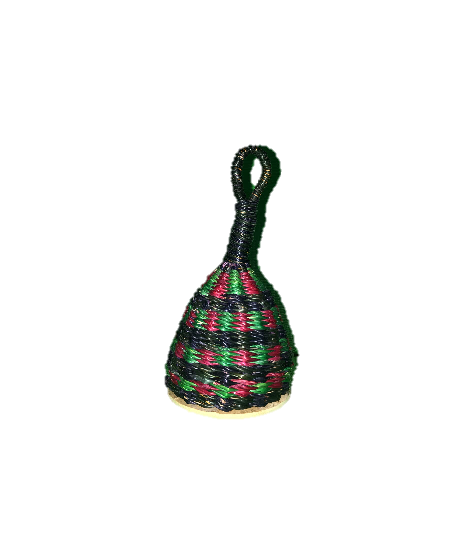5. CAXIXI  Caxixi is originally from Africa. It belongs to the group of idiophones, as it is a percussion instrument played by shaking it: the small particles inside (made by little stones or sand) hit the walls of the instrument producing sound. Depending on whether you hit the walls or the bottom, it produces different sound: softer on the sides and sharper on the bottom. Its name resembles the sound it makes. It is still played in some communities in Africa and its music is believed to call for enchanted spirits and to ward off evil ones. | 1. Where is it originally from? 2. Indonesia 3. Africa 4. Mexico 5. What type of musical instrument is (classification)? 6. Idiophone 7. Membranophone 8. Aerophone 9. Where does the name come from? 10. From african language and means rattle snake 11. From the name of the seeds inside the basket 12. From the sound it makes 13. How is it played? 14. Shaking it 15. Hitting it 16. tying it around the wristle while one dances 17. What is its social valence (in what context is it usually played)? 18. Used at weddings to bring fortune to the couple 19. Used to sing children to sleep 20. To call for enchanted spirits and ward off evil ones |
| 1. 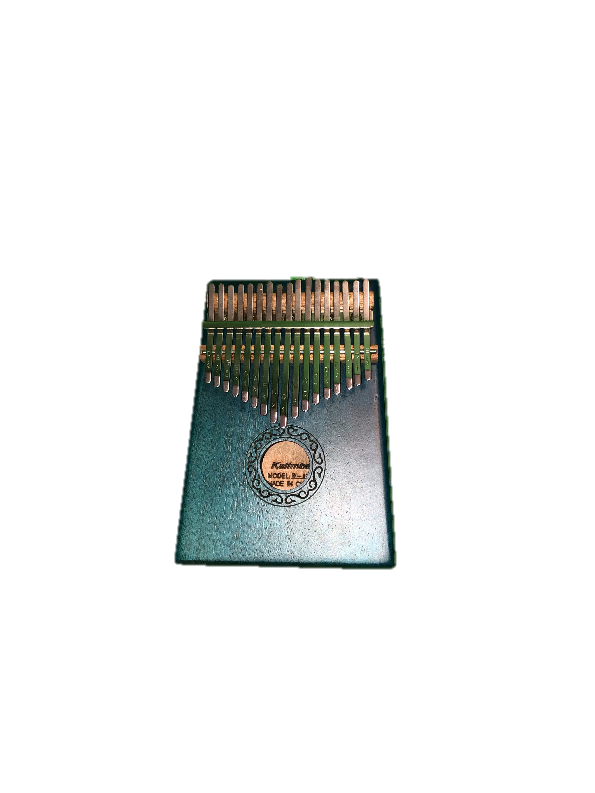KALIMBA   Kalimba is originally from Africa. It belongs to the group of idiophones, as it is a percussion instrument played by plucking the tines with the thumbs. Its name means “little music”, because of the sound it makes which is quite delicate. When out of tune, can be tuned by regulating the individual tines either a bit higher or a bit lower across the horizontal bar. It is usually associated with joyful events and played at religious ceremonies, weddings, and social gatherings to celebrate the sense of community. | 1. Where is it originally from?    1. Australia    2. Central America    3. Africa 2. What type of musical instrument is (classification)?    1. Idiophone    2. Membranophone    3. Aerophone 3. What does the name mean?    1. Hand guitar because of how it is played    2. Tiny Keyboard because of its shape    3. Little music because of its sound 4. How is it played?    1. By plucking the tines with your thumbs    2. With a pick similar to a guitar    3. By pressing on the tines like a piano key 5. What is its social valence (in what context is it usually played)?    1. Played at baby showers to symbolize new life    2. Played at funerals and wakes symbolizing transition    3. Played a religious ceremonies, weddings, and social gathering to celebrate sense of community |
| 7. PORTE-JUPE  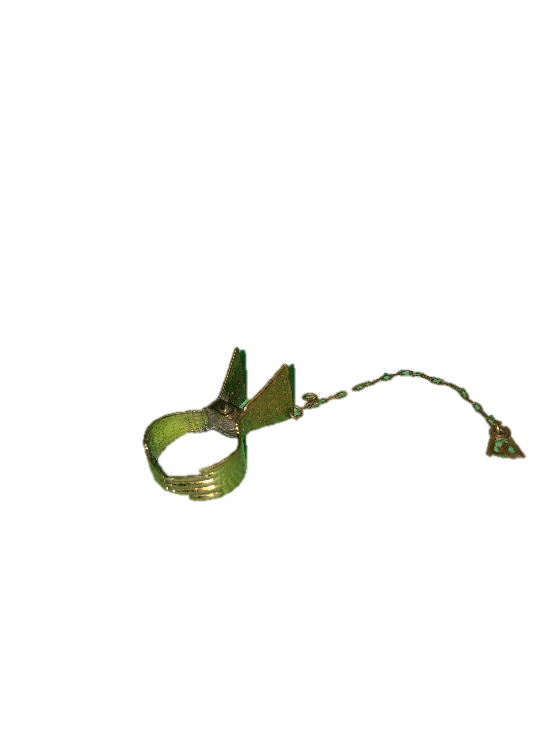  Porte-jupe used to be a female accessory popular during the Victorian age in England. It was a tong used by women to lift up their long skirt. The small ring at the top would have held a cord, ribbon, or chain to suspend the tool just below the waist. Its function was that of making women more agile when they started to engage more in outdoor activities. For this reason, it was a fashion accessory associated with emancipation of women. Its name comes from french and literally means 'dress holder'. | 1. What is its function?    1. Used as a clip for long hair    2. Used to lift long skirts    3. Used to hold multiple items 2. When was this item particularly popular?    1. Middle ages in Italy    2. Early 1900s in China    3. Victorian age in England 3. Who was used by mainly?    1. Athletes with long hair    2. Children    3. Women 4. Where does its name mean?    1. "Dress holder"    2. "Hair holder"    3. "Elegant jewellery" 5. What was its social valence?    1. It reflected style and wealth    2. It was a sign of women's emancipation    3. It was associated with elegance among athletes |
| 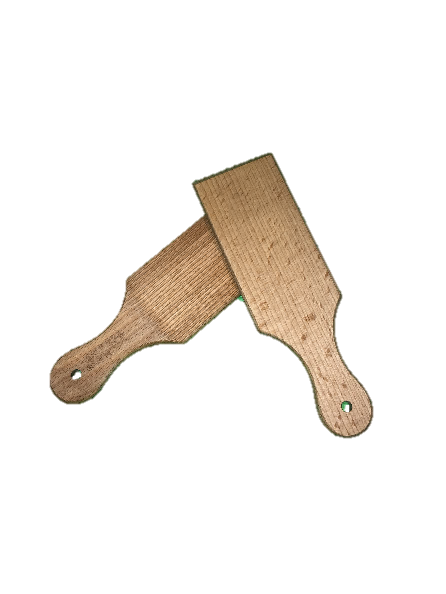8. SCOTCH HANDS  Scotch hands are kitchen tools used for making butter. They are made of wood but can also be found in metal. The external side is smooth while inside there are some small ribs. They were popular in early 1900s across the UK. They were mainly used by women and chefs, especially in big farms where they had cows and were making diary products. Their name comes from their Scottish origin and their hand-like appearance. Usually one pad stays firm while the other is moved to shape or mix salt in the butter pat. | 1. What is its function?    1. Kneading dough    2. Mixing alcoholic beverages    3. Making butter 2. When was this item particularly popular?    1. Early 1900s across the UK    2. Late 1700s in Ireland    3. 1800s throughout Europe 3. Who was used by mainly?    1. People living in industrial cities    2. Women during wedding parties    3. Women and chefs 4. Where does its name come from?    1. From its Scottish origin and hand-like appearance    2. From its usage of mixing scotch and soda    3. From its inventor 5. In which contexts was it found?    1. In wealthy houses only    2. In big farms    3. In pubs and bars |
| 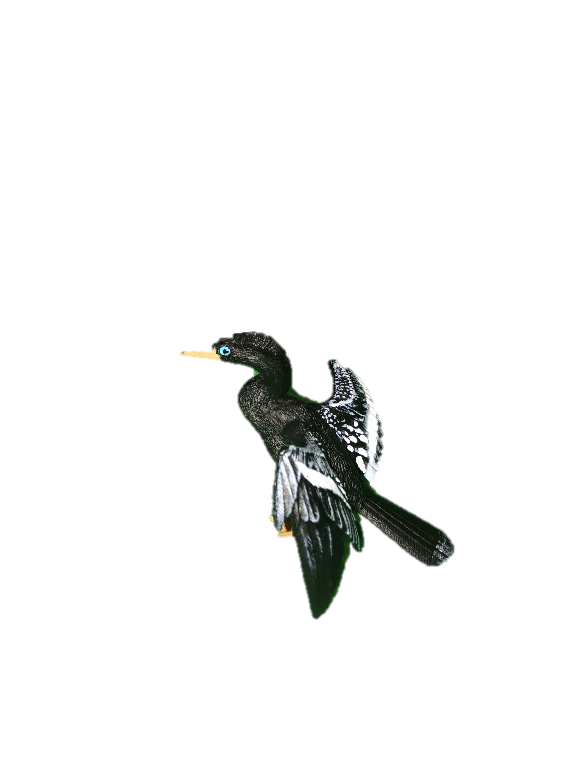9. ANHINGA  Anhinga is a bird. It lives around fresh water, including rivers and lakes. It can be found in warm parts of America (e.g. Florida, Mexico). It is also known as the water turkey, because it has a big tail similar to the one of turkeys. During breeding season, male anhinga have a blue ring around their eyes to attract female attention. It feeds on fish that it catches with its long beak. Body and feet designed to swim efficiently. It has a gland underneath its tail: this produces an oil that makes its feathers waterproof. | 1. What species is it?    1. Bird    2. Mammal    3. Fish 2. What is its habitat?    1. Forest    2. Sea    3. Fresh water 3. How is it also known as and why?    1. Air duck because of its feet    2. Water turkey because of its tail    3. Water giraffe because of its long neck 4. What does it eat?    1. Algae    2. Fish    3. Worms and insects 5. What’s its peculiarity?    1. It has special organs that allow to breathe underwater    2. It has a gland that produces an oil to waterproof its feather    3. Its saliva acts as a glue to catch little fish |
| 10. AXOLOTL  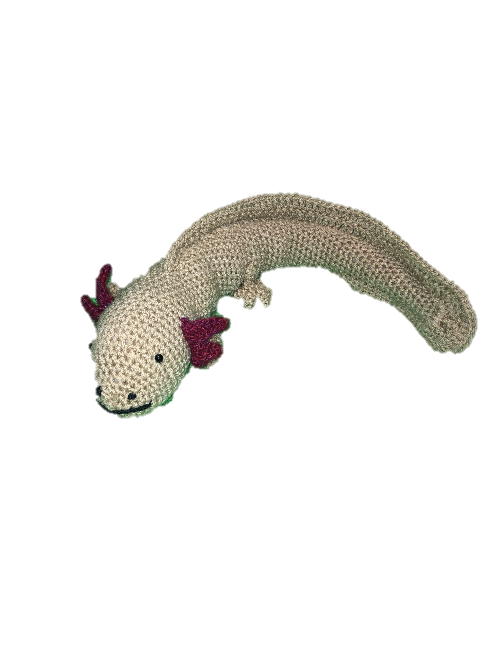  Axolotl is an amphibian which lives in the lake of Mexico. It is also known as the 'walking fish' for its way to 'walk' in water. It feeds on worms, insects and small fish, but can sometimes show cannibalism (eating another axolotl). It has a special power: it can regenerate limbs within days multiple times. For this reasons it is used a lot in staminal cell research against cancer. It reproduces asexually: male deposits the sperm into his habitat and then do a small dance to attract female so that she can find it and her eggs can be fertilised. | 1. What species is it?    1. Fish    2. Amphibian    3. Mammal 2. What is its habitat?    1. Lakes    2. Ocean    3. Rivers 3. How is it also known as and why?    1. Swimming lion because of its ray shape head    2. Sea lizard because of its body shape    3. Walking fish because of the way it moves in water 4. What does it eat?    1. Algae    2. Worms, insects and small fish    3. Dead body of big fish 5. What's its special power?    1. Can regenerate limbs    2. Can see through obstacles (rocks, other animals etc)    3. Can blend in and become invisible |
| 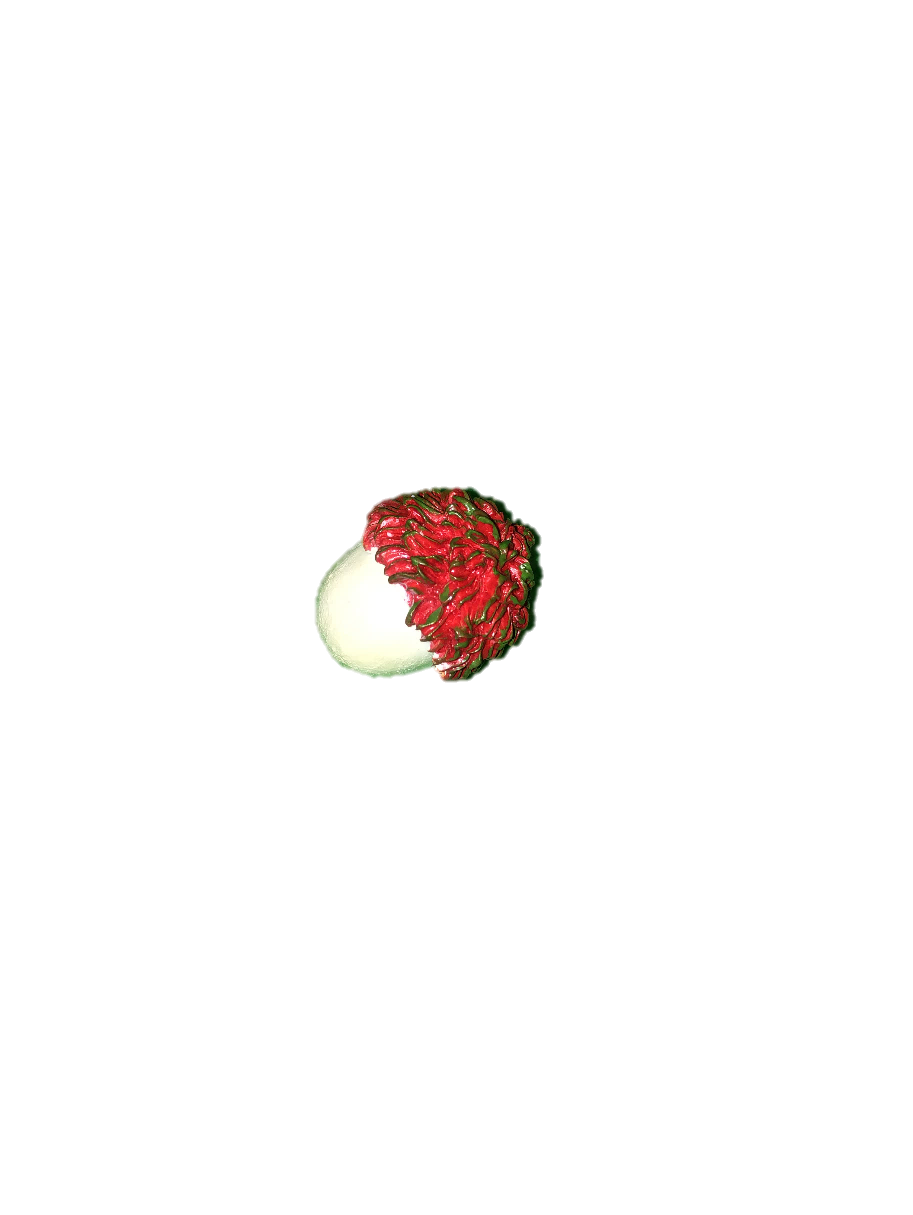11. RAMBUTAN  Rambutan is a fruit originally from southeast Asia. Because of the shape of the skin, which has thin spikes, its name means "hairy". It is all edible: it has only one big seed that can be cooked and eaten. It has a very sweet flavour which resembles that of grape, and for this reason is often used to make jams and jelly. It grows on ever-green trees, which fruit twice a year, in summer and winter. | 1. Where is it originally from?    1. Middle east    2. Southeast Asia    3. South America 2. What does the name mean?    1. Hairy    2. Spikes    3. Spider 3. What can/cannot be eaten?    1. It is completely edible a part from the central seed    2. It is completely edible including its seed    3. It is poisonous 4. What does it taste like?    1. Banana    2. Peach    3. Grape 5. In which season(s) does it grow?    1. From spirng to late summer    2. Only in summer    3. Twice per year in summer and in winter |
| 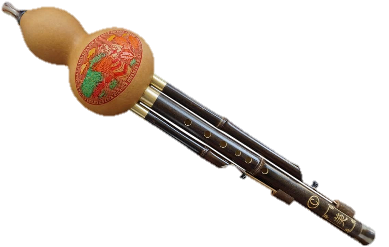12. HULUSI  Hulusi is a musical instrument originally from China. It belongs to the group of aerophone, as it is played by blowing air through it like a flute. It has three bamboo pipes, one with finger holes while the others serve to make harmonic sound effects. Sometimes one of the two pipes is clogged up and it is only ornamental. Its name means "silky gourd": 'silky' refers to the delicate sound it makes, and 'gourd' refers to its round shape on top. It is usually played by minority groups in some regions of China, Vietnam and Thailand and has generally been associated with social struggles. | 1. Where is it originally from?    1. Australia    2. China    3. Japan 2. What type of musical instrument is (classification)?    1. Aerophone    2. Idiophone    3. Membraphone 3. What does the name mean?    1. Sacred wind    2. Silky gourd    3. Long flute 4. How is it played?    1. Hitting it with a stick    2. Shaking it    3. Like a flute 5. What is its social valence (in what context is it usually played)?    1. It symbolizes minority groups    2. It represents national unity    3. It is played at big festivals |
| 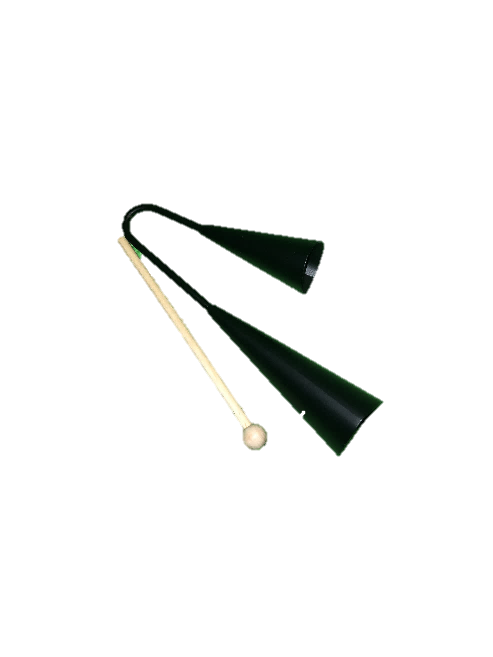13. AGOGO  Agogo is a musical instrument originally from Africa. Because of its shape, its name means ‘bells’, although the bell is in fact empty (there is no stem inside). It belongs to the group of idiophones, as it is a percussion instrument played by hitting the bells with the stick. It is played during religious ceremonies to give the right rhythm to the dance: it is believed that being in tune with the music will make it easier to be possessed by the spirits that will guide worshippers to a successful life. | 1. Where is it originally from?    1. Africa    2. China    3. Brazil 2. What type of musical instrument is (classification)?    1. Aerophone    2. Idiophone    3. Membranophone 3. What does the name mean?    1. Cones    2. Bells    3. Corn 4. How is it played?    1. Hitting it    2. Shaking it    3. Folding it 5. What is its social valence (in what context is it usually played)?    1. Played at Olympic games    2. Played during religious ceremonies    3. Played at private parties to dance |
| 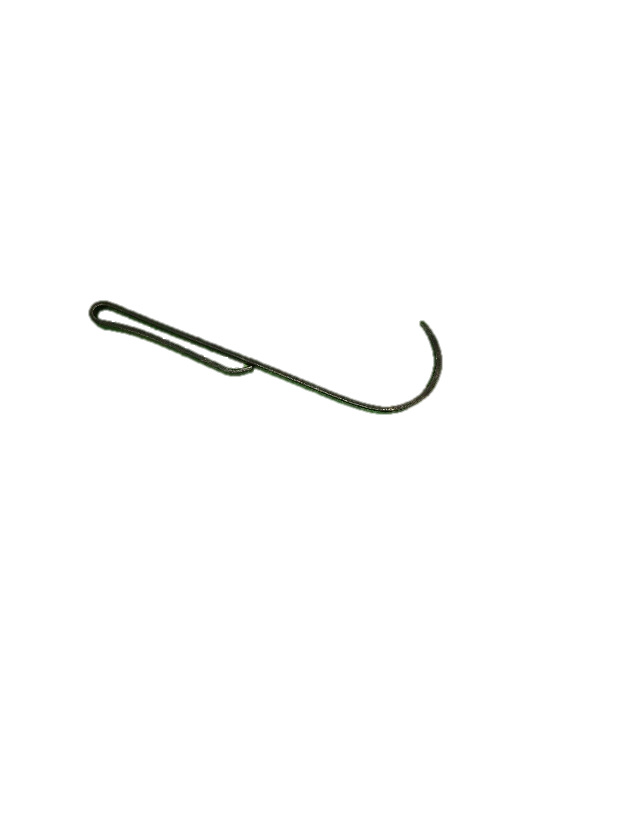14. STRIGIL  Strigil is an ancient object used during the Roman empire and ancient Greece to clean the body. It was mainly used by male athletes after their sport performance to scrape off the dirtiness from their skin before the bath. Because of its function, the name strigil literally means "scraper", from Latin. It could differ in the type of metal used and its design, depending on the social status of the owner and time period. It was often found in the tombs of important athletes to represent their social status in the afterlife. | 1. What is its function?    1. Scratch one’s back    2. Clean the body    3. Work the clay 2. When was this item particularly popular?    1. First half on 20th century    2. Ancient Egyptian empire    3. Ancient Roman and Greek times 3. Who was used by mainly?    1. Women    2. Athletes    3. Doctors 4. What does its name mean?    1. Scraper    2. Knife    3. Curved metal 5. In which contexts was it found?    1. Tombs of athletes    2. Hospitals    3. House of nobles and aristocrats |
| 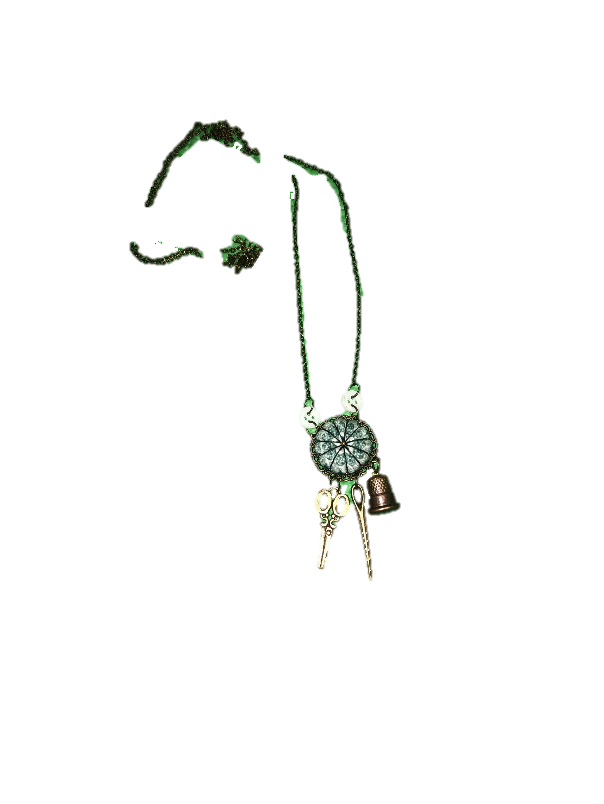15. CHATLAINE  Chatlaine is a decorative belt carrying household items including scissors, keys, needles etc. It was very popular in the middle age across Europe, and mainly wore by women. Its name comes from French and literally means "Lady of the castle". It could be made of metal, from gold to bronze or iron depending on how wealthy the owner was. As holding keys to different lockers and doors around the house, it represented a symbol of authority for the woman who wore it as other people in the houselhold had to ask her for access. | 1. What is its function?    1. Necklace which carries household items    2. Belt which carries household items    3. Decorative wristlet 2. When was this item particularly popular?    1. Late 1800s in Russia    2. 20th century in North America    3. Middle ages around Europe 3. Who was used by mainly?    1. Woman    2. Children    3. Doctors 4. Where does its name mean?    1. House care    2. Lady of the castle    3. Lease of personal belongings 5. What was its social valence?    1. Slavery within the household    2. Political responsibilities    3. Women’s authority within the household |

# Table 2 – Demographic information for NTs and clinically-diagnosed participants with ASC

a. due to technical fault, we lost demographic information from one participant. 1. (Baddeley et al., 1993) 2. (Chierchia et al., 2019) 3. (Livingston et al., 2021) 4. (Baron-Cohen et al., 2001).

|  | **Neurotypical (NT)** N=68 | | **Autistic Spectrum Condition (ASC)** N=29^a^ | | ***NT vs ASC*** | |
| --- | --- | --- | --- | --- | --- | --- |
|  | **Mean** | **SD** | **Mean** | **SD** | **t-test** | ***p-value*** |
| Age | 28.40 | 7.26 | 27.66 | 8.29 | .74 | *.68* |
| Verbal fluency (Spot the word)^1^ | 44.12 | 8.59 | 48.45 | 5.96 | -4.33 | ***.01*** |
| Non-verbal reasoning (Matrix reasoning item bank, MaRs-IB)^2^ | 61.12 | 17.14 | 63.22 | 19.04 | -2.10 | *,61* |
| Mentalising (Animated Triangle)^3^ | 9.19 | 2.09 | 9.07 | 2.02 | .12 | *.78* |
| Autistic Quotient (AQ)^4^ | 19.63 | 6.87 | 31.83 | 8.26 | -12.19 | ***<.0001*** |

# Table 3 – Sample size (N), Means and SDs for all conditions for NTs and clinically-diagnosed participants with ASC.

| *Neurotypical (NT)* | **N** | **Mean** | **SD** |
| --- | --- | --- | --- |
| Immediate: Live | 68 | 4.30 | .52 |
| Immediate: Recorded-observant | 68 | 3.99 | .64 |
| Immediate: Recorded-alone | 68 | 4.11 | .71 |
| +1week: Live | 68 | 3.90 | .78 |
| +1week: Recorded-observant | 68 | 3.66 | .76 |
| +1week: Recorded-alone | 68 | 3.72 | .76 |
| *Autistic Spectrum Condition (ASC)* | **N** | **Mean** | **SD** |
| Immediate: Live | 30 | 4.41 | .51 |
| Immediate: Recorded-observant | 30 | 4.24 | .57 |
| Immediate: Recorded-alone | 30 | 4.23 | .73 |
| +1week: Live | 30 | 3.84 | .96 |
| +1week: Recorded-observant | 30 | 3.82 | 1.04 |
| +1week: Recorded-alone | 30 | 3.77 | 1.02 |

# Table 4 – Results for clinically-diagnosed participants with ASC only.

| **Comparisons** | **F or T value** | **df** | ***p-value*** | **Partial Eta Squared** | **95% CI** | |
| --- | --- | --- | --- | --- | --- | --- |
|  |  |  |  |  | **Lower bound** | **Upper bound** |
| *Main effects* |  |  |  |  |  |  |
| Group | .61 | 1 | *.43* | .006 | -.37 | .16 |
| Learning condition | 7.06 | 2 | ***.001*** | .07 | -- | -- |
| Time | 55.61 | 1 | ***<.0001*** | .37 |  |  |
| *Interaction effects* | **F** |  |  |  |  |  |
| Group*Time | .95 | 1 | *.33* | .01 | -- | -- |
| Group*Learning condition | 1.45 | 2 | *.24* | .02 | -- | -- |
| Learning condition*Time | 1.43 | 2 | *.24* | .02 | -- | -- |
| Learning condition*Time*Group | .29 | 2 | *.75* | .003 | -- | -- |
| *Simple effects* | **t** |  |  |  |  |  |
| Live vs Recorded-observant | .18 | 1 | ***<.0001*** | -- | .09 | .28 |
| Live vs Recorded-alone | .15 | 1 | ***.004*** | -- | .05 | .29 |
| Recorded-observant vs Recorded-alone | -.03 | 1 | ***.05*** | -- | -.14 | .08 |
| Live_imm_ vs Recorded-observant_imm_ | .5.42 | 97 | ***<.0001*** | -- | .17 | .37 |
| Live_imm_ vs Recorded-alone_imm_ | 3.38 | 97 | ***.001*** | -- | .08 | .29 |
| Recorded-observant_imm_ vs Recorded-alone_imm_ | -1.44 | 97 | *.15* | -- | -.19 | .04 |
| Live_del_ vs Recorded-observant_del_ | 2.86 | 97 | ***.005*** | -- | .05 | .28 |
| Live_del_ vs Recorded-alone_del_ | 2.34 | 97 | ***.02*** | -- | .02 | .26 |
| Recorded-observant_del_ vs Recorded-alone_del_ | -.39 | 97 | *.29* | -- | -.15 | .10 |
